# Homochiral self-assembly of biocoordination polymers: anion-triggered helicity and absolute configuration inversion†
†Electronic supplementary information (ESI) available: Preparation and physical characterization data of **1^P^** and **2^M^**, additional structural description, UV-Vis and CD spectra (Fig. S1–S7), crystallographic refinement details for **1^P^** and **2^M^** (Table S1), selected bond distances and angles for **1^P^** and **2^M^** (Tables S2–S5), ESI(+)-MS and ESI(+)-MSMS spectra (Fig. S8–S11 and Schemes S1 and S2) and PXRD (Fig. S12). CCDC 1046609 and 1046610. For ESI and crystallographic data in CIF or other electronic format see DOI: 10.1039/c5sc01089f



**DOI:** 10.1039/c5sc01089f

**Published:** 2015-04-30

**Authors:** Nadia Marino, Donatella Armentano, Emilio Pardo, Julia Vallejo, Francesco Neve, Leonardo Di Donna, Giovanni De Munno

**Affiliations:** a Dipartimento di Chimica e Tecnologie Chimiche , Università della Calabria , 87036, Arcavacata di Rende , Cosenza , Italy . Email: donatella.armentano@unical.it; b Department of Chemistry , Syracuse University Syracuse , NY 13244-4100 , USA; c Departament de Química Inorgànica , Instituto de Ciencia Molecular (ICMOL) , Universitat de València , 46980 Paterna , València , Spain

## Abstract

The different natures of the weakly coordinating anions – triflate or perchlorate – in the Cu^2+^-mediated self-assembly of cytidine monophosphate nucleotide play a fundamental role in the homochiral resolution process, yielding one-dimensional copper(ii) coordination polymers of opposite helicity that can be easily inverted, in a reversible way, by changing the nature of the anion as revealed by circular dichroism experiments both in solution and in the solid state.

## Abstract 2

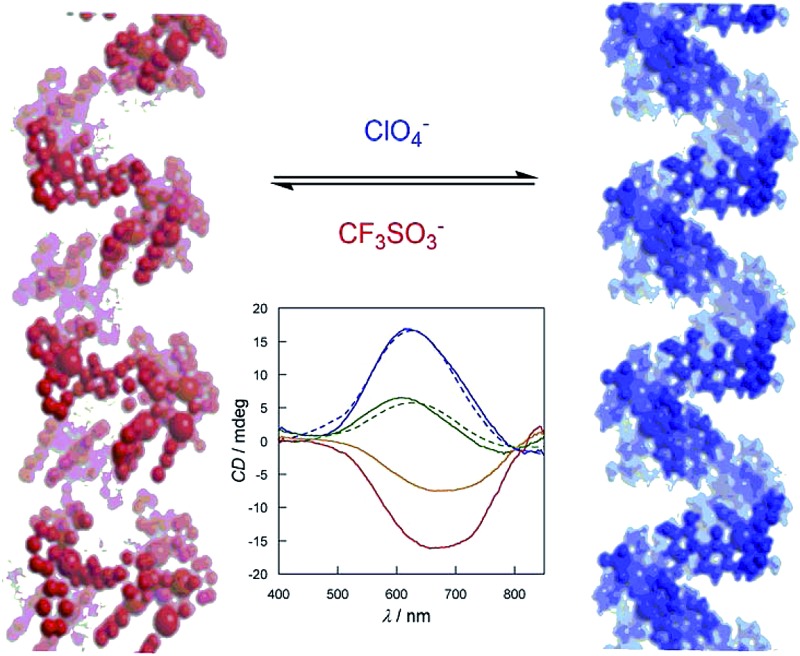
The templating roles of ClO_4_^–^ and CF_3_SO_3_^–^ allow control and reversible inversion of the chirality of nucleotide-based copper(ii) helices. These results hold great potential for developing responsive materials.

## Introduction

New generations of metal complexes containing ligands from the biological world are attracting continuous interest in the attempt to develop new materials.^1–3^ The powerful self-assembly features of biomolecules – which may have the ability to bridge metal ions with multiple possible coordination modes^2,3^ – offer the possibility to obtain both discrete zero-dimensional (0D) metal complexes and also coordination polymers of higher dimensionality (1D–3D) with fascinating architectures.^4–6^ Among the plethora of interesting properties that a coordination polymer can show, chirality has attracted intense attention from many research groups. In particular, the appearance of homochirality in biological systems, which is likely related to the origin of life,^7^ is still largely unclear. A chiral coordination complex or polymer can be obtained either in a rational way, by a judicious choice of chiral enantiopure ligands or “chiral auxiliaries” capable of transmitting their “chiral information” to the stereochemistry of the metal atoms,^8^ or serendipitously, when so-called spontaneous resolution processes^9^ occur. In this regard, interesting works have been reported recently pointing towards external factors as responsible for these spontaneous resolution processes (*e.g.* stirring,^10*a*^ rotational and magnetic forces,^10b,c^ *etc.*). Nevertheless, despite the light shed by these studies, further work is needed to fully understand this phenomenon, which could also be helpful to understand chemical processes of fundamental biological importance, such as the chirality switching experienced by DNA and proteins upon external stimuli.^11^ In this perspective, examples of metal complexes whose helicity can be inverted by external stimuli (pH, temperature, guest molecules, *etc.*) have been reported.^12^ In particular, some of them show helicity inversion in the presence of achiral anions,^13^ leading to intriguing potential applications in anion recognition.

Nucleotides, the basic constituents of nucleic acids like RNA or DNA, thus emerge as valuable ligands for the construction of a unique class of biocoordination polymers (bioCPs) with tailored architectures and tunable properties.

In the framework of our current research focused on the reactivity of first-row transition metal ions toward ligands from the biological world, such as cytidine nucleoside (H_2_cyd), we have recently shown that nucleoside-containing 3D metal complexes can be used as building blocks for the rational design of nucleoside-bridged high-nuclearity coordination compounds and high-dimensionality coordination polymers.^3^ We observed unprecedented metal-nucleoside coordination modes and excellent chiral induction, which accounted for the formation of octanuclear calixarene-like^3*a*^ as well as dodecanuclear globular-shaped complexes,^3*b*^ together with the first example of a 3D copper(ii)–cytidine coordination polymer.^3*c*^

**Scheme 1:**
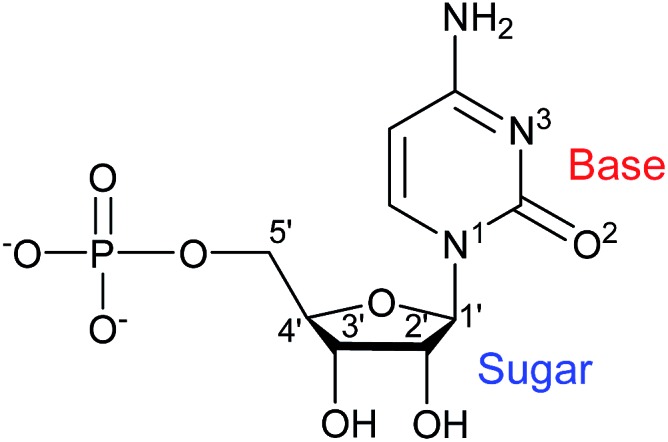
Chemical structure of the ligand CMP with selected atomic numbering.

Aiming at further exploring the potential role of this type of ligands as chiral inducers, and inspired to contribute to a better understanding of the driving forces behind supramolecular aggregations as a prerequisite for the design and construction of molecular arrays, we have more recently considered the cytidine 5′-monophosphate (CMP) nucleotide (Scheme 1).^14^ As a ligand, CMP has received relatively little consideration, always affording structurally characterized transition metal complexes that are either dimeric or, more often, polymeric in nature.^15^ On the other hand, CMP is offering good prospects as a chiral inducer in supramolecular 1D assemblies.^16^

In this paper, we show a fascinating example of anion-mediated homochiral resolution in polymeric metallo-helices, reporting on two *quasi*-identical CMP-based homochiral 1D biopolymers of opposite helicity and the respective formulas {[Cu_5_(bpy)_5_(OH)(H_2_O)_2_(CMP)_2_(ClO_4_)](ClO_4_)_4_·9H_2_O}_*n*_ (**1^P^**) and {[Cu_15_(bpy)_15_(OH)_3_(H_2_O)_7_(CMP)_6_(CF_3_SO_3_)](CF_3_SO_3_)_14_·15H_2_O}_*n*_ (**2^M^**),^17*a*^ which are built through the simultaneous self-assembly of the CMP nucleotide, the 2,2′-bipyridine (bpy) ligand and Cu(X)_2_·6H_2_O [where X = ClO_4_^–^ (**1^P^**) or CF_3_SO_3_^–^ (**2^M^**)] in aqueous solution. Interestingly, **1^P^** and **2^M^** can be rapidly interconverted by exchanging the anion, in a reversible manner (*vide infra*), with the corresponding inversion of the copper(ii) absolute configuration and helicity.

## Results and discussion

**Fig. 1:**
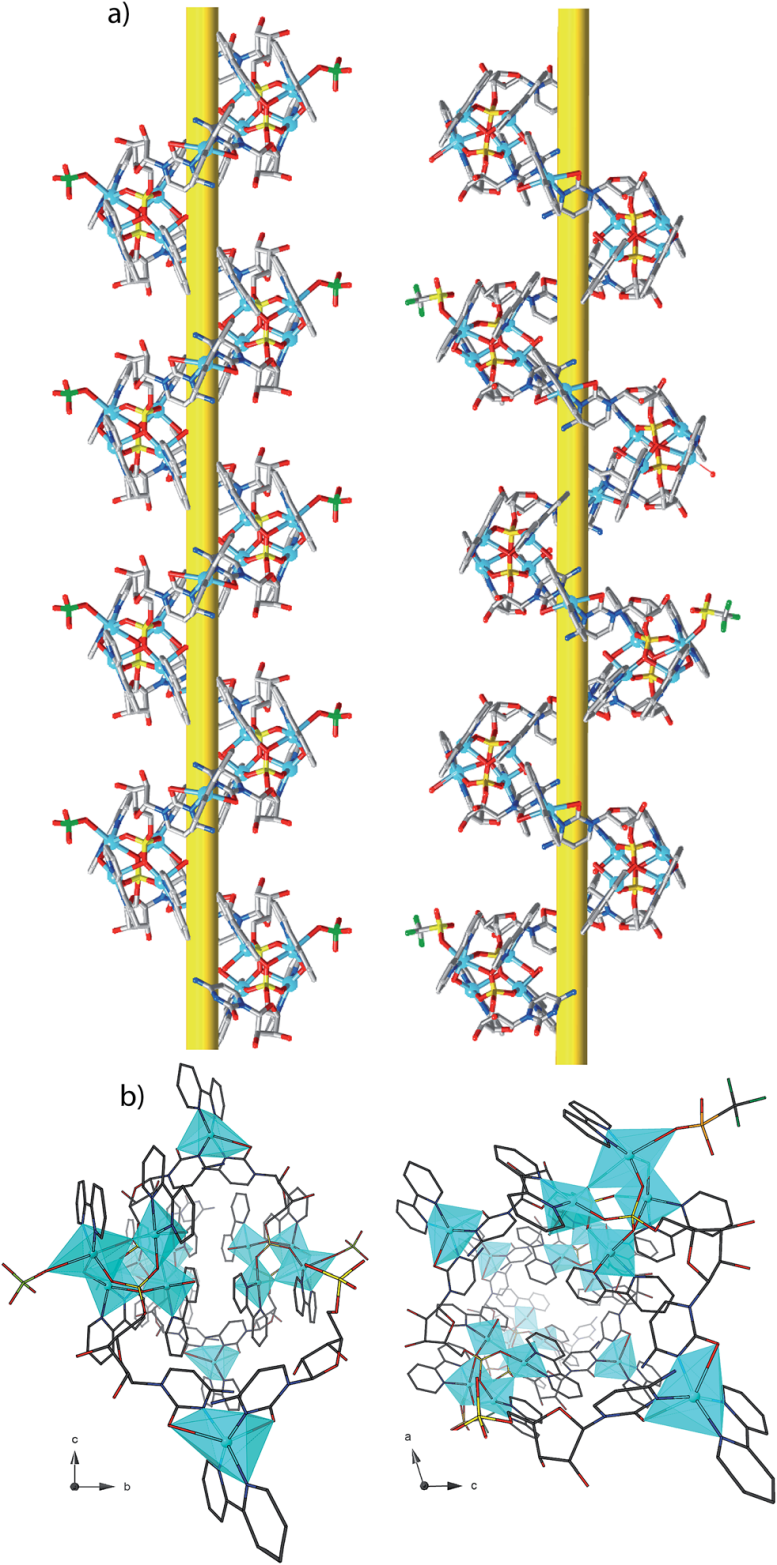
Side view (a) and top view (b) of the cationic copper(ii) chains of **1^P^** (left) and **2^M^** (right).

**Fig. 2:**
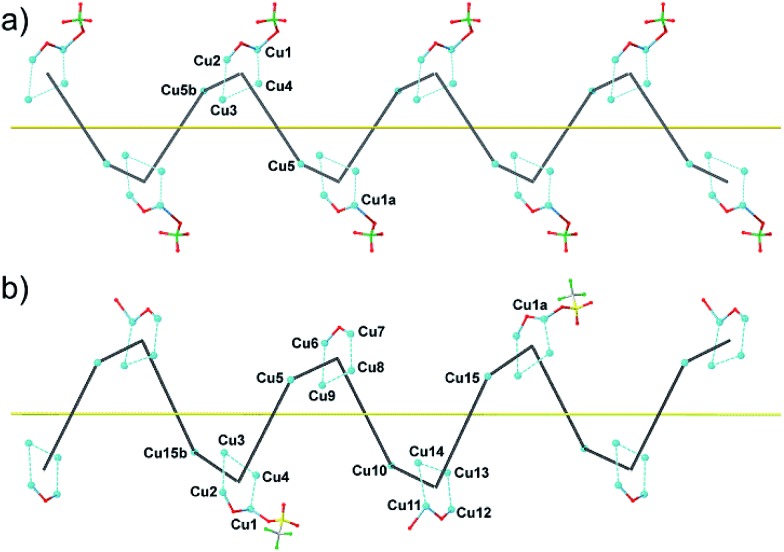
Schematic view for **1^P^** (a) and **2^M^** (b) with the metal atom numbering scheme [symmetry code (**1^P^**): *a* = *x* – 1/2, –*y* + 3/2, –*z* + 2; *b* = *x* + 1/2, –*y* + 3/2, –*z* + 2; (**2^M^**): *a* = –*x* + 1, *y* – 1/2, –*z* + 1; *b* = –*x* + 1, *y* + 1/2, –*z* + 1].

**1^P^** and **2^M^** crystallize in the chiral space groups *P*2_1_2_1_2_1_ and *P*2_1_ of the orthorhombic and monoclinic systems, respectively, their absolute configuration being reliably assigned. The structure of **1^P^** consists of single-stranded helices containing the repeating unit [Cu_5_(bpy)_5_(H_2_O)_2_(OH)(CMP)_2_(ClO_4_)]^4+^ (including a single, weakly-coordinating ClO_4_^–^ ion) (Fig. 1, left and Fig. 2), perchlorate counterions and a large amount of lattice water molecules. On the other hand, fifteen crystallographically independent copper atoms are present in the repeating cationic unit of **2^M^**, which could be alternatively formulated^17*a*^ as {[Cu_5_(bpy)_5_(H_2_O)_2_(OH)(CMP)_2_(CF_3_SO_3_)][Cu_5_(bpy)_5_(H_2_O)_3_(OH)(CMP)_2_][Cu_5_(bpy)_5_(H_2_O)_2_(OH)(CMP)_2_]}^14+^ (see Fig. 1, right and Fig. 2). The single-stranded helices are arrayed in a right-handed (**1^P^**) or left-handed (**2^M^**) fashion, with similar helical pitches [16.431 Å (the *a* axis value) for **1^P^** and 16.538 and 16.785 Å (*ca.* 1/3 of the *b* axis value) for **2^M^**].^17*b*^

**Fig. 3:**
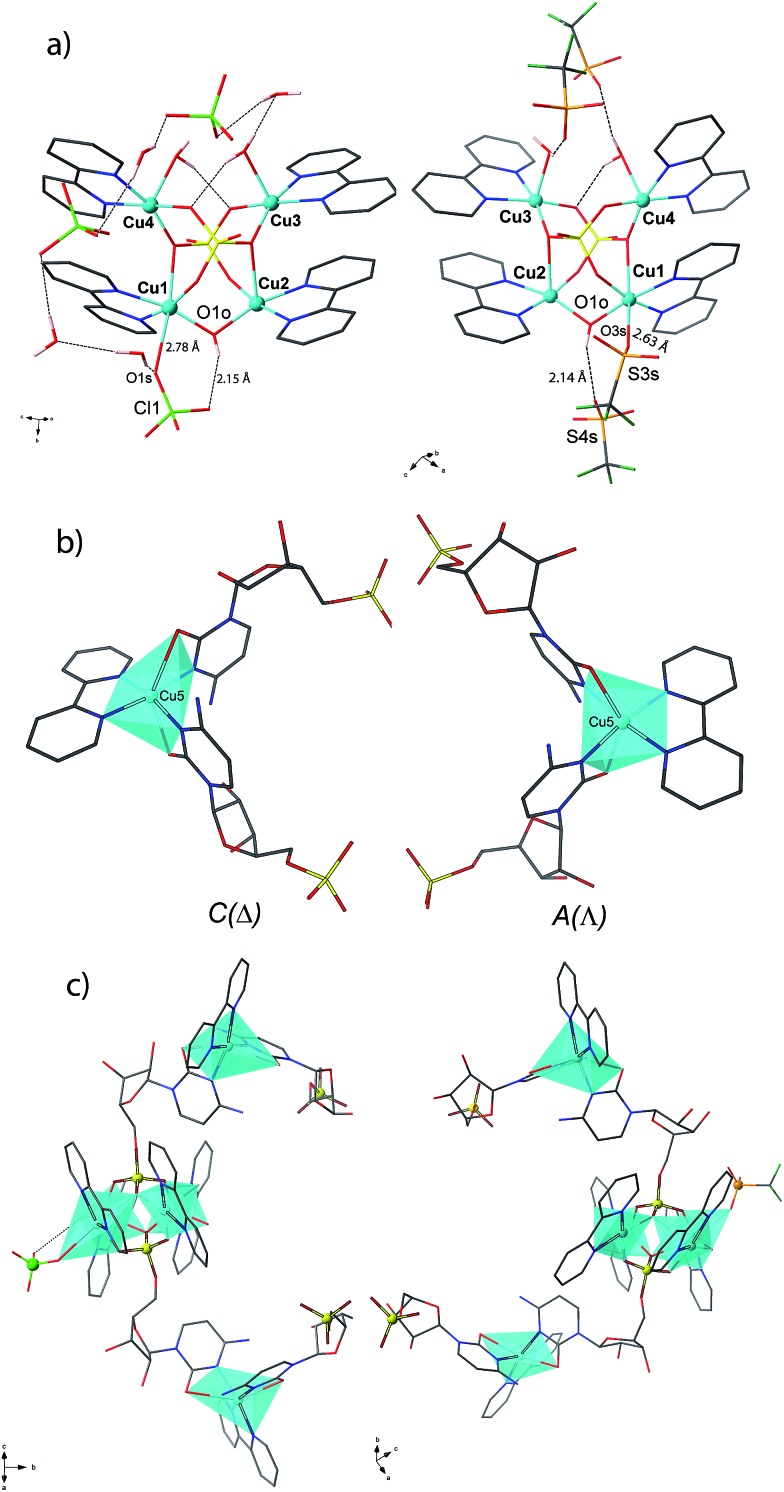
Tetranuclear cores (a), Cu(ii) connectors (b) and a portion of the single-stranded helix of alternating tetranuclear cores (c) for **1^P^** (left) and **2^M^** (right). *C*/*A* are general chiral descriptors while *Δ*/*Λ* refer to axial chirality.^19^

Each helix of **1^P^** and **2^M^** contains CMP ligands coordinated through the oxygen atoms of the phosphate groups and *via* N(3) and the exocyclic O(2) of the nucleobase as bridges (Fig. 3). Pairs of μ_4_-phosphate groups connect four copper(ii) ions, giving rise to butterfly-shaped *tetranuclear cores* of the type [Cu_4_(μ_4_-PO_4_)_2_(μ-OH)], also supported by a bridging hydroxo group (Fig. 2, 3a and S1†). The remaining copper atoms, chelated by the cytosine base of the nucleotides, (Fig. 2 and 3b), constitute the *connectors* of the CMP ligands, *i.e.* the chiral inducers of the overall helical structure. In fact, they exhibit an octahedral geometry in both **1^P^** and **2^M^** but with opposite *C*(*Δ*) or *A*(*Λ*) propeller chirality.^18,19^ Since the role of such *connectors* is pivotal, each helix consists of a single strand of alternating *tetranuclear cores* and *chiral connectors* (Fig. 3c), leading to helices with *P* (**1^P^**) or *M* (**2^M^**) chirality.

Interestingly, within the cores only one of the four metal ions is further anion-linked achieving an octahedral geometry. In fact, while in **1^P^** merely the perchlorate anion is weakly coordinated to one copper atom in four, in **2^M^** competition between the water solvent and the triflate ion actually leads to three different tetranuclear cores. Two of them [core I (Fig. 3b, right and S2†) and core III (Fig. S4†)], present an *extra* coordination site either occupied by a triflate anion or by a water molecule. Finally, in the third one (cluster II in Fig. S3†), neither anions nor solvent molecules are linked to the would-be octahedral copper, further proving the lability of the *extra* anion/solvent coordination. At this point, one may immediately notice that contrary to the general expectation based on the slightly lower coordinating ability of the perchlorate ion,^20^ coverage of the bound triflate along the polymeric chain is consistently smaller. Thus, fewer anions are directly linked to the metal centres of the helix in **2^M^** with respect to **1^P^** (Fig. 1 and 2), with both electronic and structural consequences.

In the search for the driving force behind the opposite supramolecular helical chirality in **1^P^** and **2^M^**, we found that the ClO_4_^–^ anion coordinated in **1^P^**, is hydrogen-bonded to the bridging hydroxo group within the tetranuclear unit (Fig. 3a, left), suggesting that the copper ions showing a *C* chirality can be stabilized more effectively than the corresponding *A* form (Fig. 3, right). On the contrary, the corresponding triflate-coordinated copper ion in **2^M^** (cluster I, Fig. 3b and S2†), shows an *A* configuration.

In general, the coordinated anions in **2^M^** are not at all or scarcely involved (see Fig. S4†) in intramolecular H-bonds with the hydroxo group of the tetranuclear core. In cluster I, for instance, the hydroxo group is engaged with a free CF_3_SO_3_^–^. Thus our hypothesis is that the different nature, size and shape of the CF_3_SO_3_^–^ anion represent the major reasons for the *A* absolute configuration of the anion-coordinated metal centres in **2^M^**. The geometry around the sulphur atom, combined with the potential steric clash of the CF_3_ group with the closest free ribose moiety of CMP, most likely causes a destabilization of the *C* form. For that reason the coordinated CF_3_SO_3_^–^ is not involved in intramolecular H-bonding, and the hydroxide group of the tetranuclear core is at this time engaged by a free CF_3_SO_3_^–^.

Remarkably, all metal ions within the *tetranuclear cores* and *connectors*, exhibit the same chirality as the anion-coordinated metal center (*i.e.* Cu(i)), confirming thus the transmission of the chiral properties induced by the anions to the coordinated metal center through weak interactions. Each homochiral asymmetric fragment of the helices [
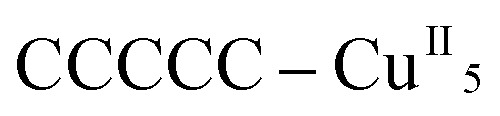
 (**1^P^**) and 
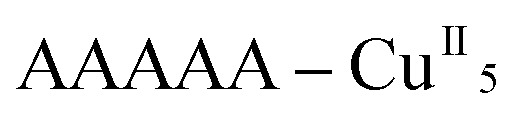
 (**2^M^**)] then has opposite chirality. These structural features suggest that the anions play a key role in the self-assembly process that leads to the chain formation with opposite handedness.

The pseudo-cylindrical helices reveal further different structural features depending on the anions, forming a pillar of metal ions with the shortest intrahelical Cu_cores_···Cu_connectors_ separations varying in the range 10.21–12.00 Å in **1^P^** and 8.78–12.53 Å in **2^M^**, with a clear putative role of the different anions in the induction of prominently different arrangements of the copper ions along the helical chains. Notably, in **1^P^** the sugar moieties of the CMP ligands exhibit the C(3′) – *exo* conformation while in **2^M^** they show the C(2′) – *endo* conformation. The conformation of C(4′)–C(5′) is *gauche*–*gauche* for both compounds. The chiral centers of the CMP ligand C1′, C2′, C3′ and C4′ in the helices have the configurations *R*, *R*, *S*, *R*.

The packing of **1^P^** and **2^M^** produces small voids inside and outside each chain, (Fig. S5†) where the lattice water molecules and ClO_4_^–^ (**1^P^**) or CF_3_SO_3_^–^ (**2^M^**) anions reside as blocks of a 3D H-bonded grid. The potential solvent and anion accessible areas around the chains account for 31.8% (**1^P^**), and 37.7% (**2^M^**) of the unit cell volume.

**Fig. 4:**
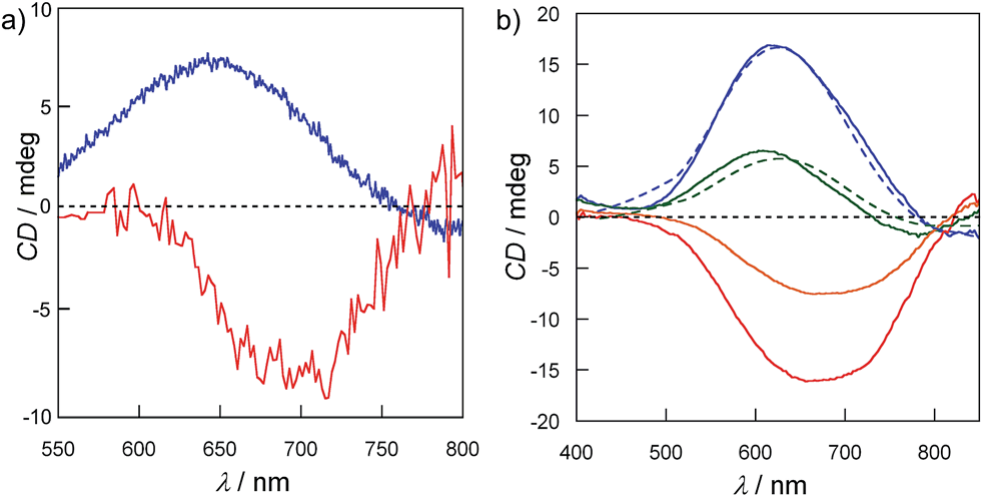
(a) CD spectra of **1^P^** (blue) and **2^M^** (red) in the visible region as KBr pellets (*ca.* 1 mg of complex/100 mg of KBr). (b) Evolution of the CD spectra of a H_2_O/CH_3_CN (1 : 1 v/v) solution of **1^P^** (blue line) (10^–3^ M) upon the addition of LiCF_3_SO_3_ (0 eq., blue line; 2 eq. green line; 4 eq. orange line; 6 eq. red line) followed by the addition of LiClO_4_ (2 eq. dashed green line; 4 eq. dashed blue line).

In order to confirm the enantiopurity of the sample in a given synthesis, and also to ensure the reproducibility of the results, solid circular dichroism (CD) experiments were carried out for crystals of five different syntheses. The solid CD spectra of **1^P^** and **2^M^** in the visible region (Fig. 4a) confirm the absolute configuration of the chiral metal centers. An almost mirror image^21^ can be observed for **1^P^** and **2^M^**. Thus, **1^P^** exhibits a broad maximum positive Cotton effect at 644 nm whereas **2^M^** exhibits a maximum negative Cotton effect at *ca.* 690 nm with a similar intensity to that of **1^P^**. Both bands are attributed to d–d transitions as a result of the chirality induced effect on the copper(ii) ions. These opposite Cotton effects further confirm that the presence of the different ClO_4_^–^ or CF_3_SO_3_^–^ anions induces opposite chirality on the metal centers of **1^P^** and **2^M^**. Fig. S6† shows identical Cotton effects for both **1^P^** and **2^M^** in the UV region, that is, negative and positive Cotton effects at *ca.* 220 and 280 nm, respectively. These former bands have already been reported for the CMP ligand,^15^ and, ultimately, confirm the presence of d-ribose in its enantiopure natural form in both **1^P^** and **2^M^**. H_2_O/CH_3_CN (1 : 1 v/v) solution CD experiments, which are depicted in Fig. S7,† show similar positive and negative Cotton effects, confirming that homochiral species with opposite absolute configurations for the copper(ii) ions are also present in solution.

Challenged by these results, and in order to check the reversible nature of this anion-mediated chiral resolution process, we monitored the anion exchange in H_2_O–CH_3_CN mixture (1 : 1 v/v) solutions of **1^P^** and **2^M^** by measuring the CD spectra after controlled additions of LiCF_3_SO_3_ and LiClO_4_ salts, respectively. Fig. 4b shows the evolution of the CD spectra of a **1^P^** solution in H_2_O/CH_3_CN (1 : 1, v/v) with increasing [CF_3_SO_3_^–^]. The intensity of the initial positive Cotton effect decreases with the addition of LiCF_3_SO_3_ to become finally negative. Then, the reversible nature of the process was verified when the positive Cotton effect was recovered by adding LiClO_4_ (dashed lines in Fig. 4b). This reversible and fast (going to completeness in a few minutes) switching in solution, achieved by changing the nature of the anion, is most likely due to the labile coordination of both the ClO_4_^–^ and CF_3_SO_3_^–^ anions to the Cu(ii) centre of the cores; thus they are undoubtedly exchanged.

In order to achieve a better understanding of the stable oligomeric species in solution – whose absolute configuration can be dynamically switched by changing the nature of the achiral anion as shown by the CD spectra – ESI-MS and related tandem mass spectroscopy (MS/MS) experiments were carried out for solutions of **1^P^** and **2^M^**. The presence of stable oligomers in solution can be inferred by the electrospray experiments, which show dimeric species for **1^P^** and both trimeric and dimeric ones for **2^M^** (see Schemes S1 and S2 and Fig. S8–S11†).

These results prompted us to speculate that the switching of the absolute configuration of the copper(ii) ions of the oligomeric species lies at the origin of the helix inversion in the final solid-state coordination polymers. In order to confirm our hypothesis, we did a final test. Crystals of **1^P^** were dissolved in water/acetonitrile, an excess of LiCF_3_SO_3_ salt was added to the solution, and the product was allowed to precipitate. The powder X-ray diffraction pattern of the polycrystalline solid that appeared is shown in Fig. S12† confirming the crystallization of the pure enantiomer **2^M^**. The application of the same procedure to crystals of **2^M^** gave a similar outcome, as reported in the ESI† material.

## Conclusions

In summary, we report herein a remarkable example of anion-triggered homochiral induction that yields two copper(ii) 1D coordination polymers of opposite chirality depending on the anion used: ClO_4_^–^ (**1^P^**) or CF_3_SO_3_^–^ (**2^M^**). Interestingly, we have also shown that the absolute configuration at the copper(ii) ions and the sense of the helix (or at least a fragment of it), can be dynamically switched – in a similar manner to what happens in biological DNA – by using achiral anions of different natures. The labile anion coordination accounts for the switching of the copper ion configuration and the consequent helicity inversion of the entire chain (or oligomer). In these blocks the orientation of the nucleobase rings is such as to place the carbonyl pointing toward the copper atoms, with its oxygen atom occupying the elongated axial positions, and it is therefore easily flexible and removable on demand.

Although a few examples of polynuclear complexes and coordination polymers exhibiting switching of chirality triggered by achiral anions have been reported,^12,13^ evidence of coordination *bio*-helix inversion is unprecedented. Achieving control over the structure of a nucleotide-based helix holds great potential for developing stimuli-responsive materials matching the level of sophistication of biological systems, with potential applications in memory devices, biomimetic materials, specific ion sensors and molecular recognition.^12^ In order to get deeper insight into the rational design of chiral systems, current efforts are devoted to further investigating the putative templating role of these anions in novel examples of chiral coordination polymers with other nucleotide-based ligands. Exploration of the coordination flexibility of the CMP and other nucleotides to yield chiral metal-organic frameworks (MOFs) is a second important goal of our research.
