# Supplementary material for: Homochiral self-assembly of biocoordination polymers: anion-triggered helicity and absolute configuration inversion
Source: Chem Sci. 2015 Apr 30;6(7):4300–5. doi: 10.1039/c5sc01089f (PMC5707485; doi:10.1039/c5sc01089f)
Supplement: Supplementary file 1 [file SC-006-C5SC01089F-s001.pdf]

Electronic Supplementary Material (ESI) for Chemical Science.
This journal is © The Royal Society of Chemistry 2015

Electronic Supporting Information (ESI):

Homochiral Self-Assembly of Biocoordination Polymers: Anion-Triggered Helicity and Absolute Configuration Inversion

Nadia Marino, Donatella Armentano, Emilio Pardo,* Julia Vallejo, Francesco Neve, Leonardo Di Donna and Giovanni De Munno*

Experimental Section

Materials. All chemicals were of reagent grade quality. They were purchased from commercial sources and used as received.

Preparation of $[Cu_5(bpy)_5(OH)(H_2O)_2(CMP)_2(ClO_4)](ClO_4)_4 \cdot 9H_2O$ ($\mathbf{1^P}$): The copper(II) chain $\mathbf{1^P}$ was prepared in very good yields from the reaction of stoichiometric amounts of $Cu(ClO_4)_2.6H_2O$, bpy and the corresponding enantiopure CMP ligand, commercially available (1:1:1 metal to ligands molar ratio) in water solutions. X-ray quality blue rhombuses prisms of $\mathbf{1^P}$ were obtained by slow evaporation of H_2O/EtOH (2:1 v/v) mixtures. Yield: 70%. Elemental analysis calcd (%) for $C_{68}H_{87}Cl_5Cu_5N_{16}O_{48}P_2$ ($\mathbf{1^P}$): C 33.29; H 3.57; N 9.13. Found: C 33.59; H 3.47; N, 9.10. IR (KBr): ν = 1688vs, 1641vs and 1370s cm^{-1} (C=O) from CMP and 1474s and 1440 cm^{-1} (C=C) from bpy.

Preparation of $\{[Cu_{15}(bpy)_{15}(OH)_3(H_2O)_{6.7}(CMP)_6(CF_3SO_3)_{1.3}](CF_3SO_3)_{13.7} \cdot 15H_2O\}_n$ ($\mathbf{2^M}$): The copper(II) chain $\mathbf{2^M}$ was prepared from the reaction of stoichiometric amounts of $Cu(CF_3SO_3)_2 \cdot 6H_2O$, bpy and the corresponding enantiopure CMP ligand, commercially available (1:1:1 metal to ligands molar ratio) in water solutions. X-ray quality light blue rhombuses prisms of $\mathbf{2^M}$ were obtained by slow evaporation of H_2O/EtOH (2:1 v/v) mixtures. Yield 65%. Elemental analysis calcd (%) for $C_{219}H_{238.4}S_{15}Cu_{15}F_{45}N_{48}O_{117.7}P_6$ ($\mathbf{2^M}$): C 33.29; H 3.04; N 8.51. Found: C 33.40; H 3.12; N, 8.52. IR (KBr): ν = 1681vs, 1637vs and 1383s cm^{-1} (C=O) from CMP and 1474s and 1440 cm^{-1} (C=C) from bpy.

Physical Techniques. Elemental analyses (C, H, N) were performed at the microanalysis service of the Dipartimento di Chimica e Tecnologie Chimiche, of the Università della Calabria (Italy). The FTIR spectra were recorded on a Nicolet-6700 spectrophotometer as KBr pellets. The UV-Vis spectra were recorded on a Nicolet Evolution 600 spectrophotometer. Natural circular dichroism (NCD)

curves were recorded using a Jasco model J-810 spectropolarimeter. The powder X-ray diffraction patterns at room temperature were collected on a Bruker AXS General Area Detector Diffraction System with Cu-Kα radiation (λ = 1.54056 Å). Mass spectrometric analysis was performed on a triple quadrupole TSQ Quantum Vantage (Thermo Fisher Scientific) mass analyzer fitted with a heated electrospray ionization (HESI II) source operating in positive ion mode. The following working conditions were applied: spray voltage: 2.8 kV; vaporizer and capillary temperature: 275 and 200 °C, respectively; sheath and auxiliary gas at 40 and 50 arbitrary units (a.u.), respectively; the collision gas was Argon used at a pressure in the collision cell (Q2) of 1.5 mTorr and the mass resolution at the first (Q1) and third (Q3) quadrupole was set at 0.7 Da at full width at half maximum (FWHM). S-lens RF amplitude was kept at 183 V while collision energy (CE) for MSMS spectra was kept at 15 eV. The samples concentration was 30μg/mL.

Crystal Structure Data Collection and Refinement. Crystal Structure Data Collection and Refinement.

X-ray crystallographic data for $\{[Cu_5(bpy)_5(H_2O)_2(OH)(CMP)_2(ClO_4)](ClO_4)_4 \cdot 9H_2O\}_n$ (**1^P**) and $\{[Cu_{15}(bpy)_{15}(H_2O)_{6.7}(OH)_3(CMP)_6(CF_3SO_3)_{1.3}](CF_3SO_3)_{13.7} \cdot 15H_2O\}_n$ (**2^M**) were collected with a Bruker Apex Duo-diffractometer coupled to a Bruker-AXS Apex II CCD detector with graphite-monochromated Cu Kα radiation (λ = 1.54178 Å) at 90(2) K and a Bruker-Nonius X8 APEXII CCD area detector diffractometer using graphite-monochromated Mo-Kα radiation (λ = 0.71073 Å) at room temperature, respectively, performing φ-and ω-scans. Suitable crystals of approximate dimensions 0.18 x 0.15 x 0.15 mm (**1^P**) and 0.30 x 0.28 x 0.24 mm (**2^M**) were selected for data collection. Data reduction and multi-scan absorption corrections were performed using SAINT[1] and SADABS.[2] The structures were solved by direct methods using SHELXS and refined against F^2 on all data by full-matrix least squares with either SHELXL-2013[3] (**1^P**) or SHELXL-97[4] (**2^M**). Graphical manipulations were performed with the XP utility of the SHELXTL system and the CrystalMaker[5]

software. Crystal data for **1^P^** and **2^M^** are summarized in Table S1. CCDC reference numbers are CCDC 1046609 and 1046610, respectively.

Refinement details for 1$^{\mathbf{P}}$: All non-hydrogen atoms were refined anisotropically. The hydrogen atoms of the bipy and CMP ligands were set in calculated positions and refined using a riding model, except those on the N(4) atom of the cytosine nucleobases, which were located on the ΔF map and refined with distance restraints instead. The hydrogen atom on the hydroxo group in the tetranuclear core and those on the water molecules (both coordinated and not-coordinated) were located on the ΔF map and refined with distance restraints, with thermal factors fixed to 1.5 times the *U* value of the oxygen atom they are linked to. The perchlorate ions were refined with the aid of similarity restraints on 1,2- and 1,3-distances and rigid-bond restraints.[6]

Refinement details for 2$^{\mathbf{M}}$: All non-hydrogen atoms were refined anisotropically, except the O(18w)-O(22w) oxygen atoms, belonging to disordered water molecules of crystallization. The bipy ligands were refined using geometrical constraints to help improving the data to parameter ratio. The hydrogen atoms of the bipy and CMP ligands were set in calculated positions and refined using a riding model, except those on the N(4) atom of the cytosine nucleobases, which were located on the ΔF map and refined with distance restraints instead. The hydrogen atom on the bridging hydroxo group and those on the coordinated water molecules O(1w)-O(6w) in the three tetranuclear cores of **2**$^{\mathbf{M}}$ were at first placed in calculated positions following the direction of plausible hydrogen bonds, then shortly refined with distance restraints and thermal factors fixed to 1.5 times the *U* value of the oxygen atom they are linked to, then fixed. The hydrogen atoms on the water molecules of crystallization were not placed. The large amount of free triflate counterions was found affected by some disorder. The disordered triflate ions were refined with the aid of similarity restraints on 1,2- and 1,3-distances and rigid-bond restraints. One of such disorder was found to affect the tetranuclear core indicated as cluster III (see Figure S4). In our best model for **2**$^{\mathbf{M}}$, in fact, a water molecule competes against a triflate anion for the coordination to the Cu(11) atom. This particular triflate anion appears thus statistically disordered; we modeled it over two sites [S(15s), O(43s), O(44s),

O(45s), C(15s), F(43s), F(44s), F(45s) and S(16s), O(46s), O(47s), O(48s), C(16s), F(46s), F(47s), F(48s) sets of atoms], one in the Cu(11) coordination sphere and one out: the relative occupancy of each disorder component was refined freely while constraining the sum of the occupancies to unity.[6] The competing water molecule [O(7w)], included in the disorder model, was refined freely while constraining the sum of its own occupancy and that of the coordinated triflate ion to unity. This model provided atom Cu(11) in cluster III being coordinated either to a water molecule or a triflate ion with a statistics of about 70% vs. 30%, respectively.

1. *SAINT*, version 6.45; Bruker Analytical X-ray Systems: Madison, WI, **2003**.
2. Sheldrick, G. M. *SADABS,* University of Göttingen, **1996**.
3. Sheldrick, G. M. *Acta Crystallogr.* **2008**, *A64*, 112-122.
4. Shelxl-97, Bruker Analytical X-ray Instruments, Madison, WI, 1997.
5. D. Palmer, CRYSTAL MAKER, Cambridge University Technical Services, C. No Title, 1996.
6. Müller, P. *Crystallography Reviews* **2009**, *15*, 57-83.

Table S1. Summary of crystallographic data for 1^P and 2^M

Formula	$C_{68}H_{87}Cl_5Cu_5N_{16}O_{48}P_2$	$C_{219}H_{238.34}Cu_{15}F_{45}N_{48}O_{117.67}P_6S_{15}$
λ / cm^{-1}	1.54178 (CuKα)	0.71073 (MoKα)
M / g mol^{-1}	2453.53	7900.37
Crystal system	orthorhombic	monoclinic
Space group	$P2_12_12_1$	$P2_1$
a / Å	16.4315(12)	17.821(2)
b / Å	18.8700(14)	49.945(5)
c / Å	30.286(4)	18.529(2)
β / °		108.269(6)
V / Å^3	9390.5(12)	15661(3)
Z	4	2
ρ_{calc} / g cm^{-3}	1.735	1.675
μ / mm^{-1}	3.827	1.250
T / K	90(2)	293(2)
F(000)	5004	7999
Crystal size	0.18 x 0.15 x 0.15	0.32 x 0.28 x 0.20
Theta range for data collection	2.76 to 68.24°.	2.76 to 54.00°.
Index ranges	-19<=h<=19,-21<=k<=22,-35<=l<=36	-22<=h<=22, -63<=k<=63, -23<=l<=23
Reflect. collcd.	73258	560634
Independent reflections	16885 [R(int) = 0.0385]	67880 [R(int) = 0.0588]
Completeness to ϑ = 25.00°	100 %	100 %
Data / restraints / parameters	16885 / 335 / 1379	67880 / 853 / 3948
$R1^a$ [$I > 2\sigma(I)$] (all data)	0.0291 (0.0318)	0.0514 (0.0621)
$wR2^b$ [$I > 2\sigma(I)$] (all data)	0.0710 (0.0723)	0.1454 (0.1564)
Goodness-of-fit^c	1.021	1.027
Absolute structure parameter (Flack)	0.015(12)	0.015(5)
Largest diff. peak and hole / e Å^{-3}	0.973 and -0.495 e.Å-3	1.786 and -1.587 e.Å-3

^{a} $R_1 = \sum(|F_o| - |F_c|)/\sum|F_o|$. ^{b} $wR_2 = [\sum w(F_o^2 - F_c^2)^2/\sum w(F_o^2)^2]^{1/2}$. ^{c} $S = [\sum w(|F_o| - |F_c|)^2/(N_o - N_p)]^{1/2}$.

Further structural details

Hereunder have been reported details concerning the butterfly tetranuclear cores of copper ions for **1^P^** and **2^M^**.

Within the tetranuclear cores in $\mathbf{1^P}$ three of the four copper ions exhibit a slightly distorted square pyramidal environment the fourth being six-coordinated in an elongated distorted octahedral environment (Fig. S1). In $\mathbf{2^M}$ there are three crystallographically not equivalent tetranuclear cores (I-III), one consisting of all four Cu^{II} ions in a slightly distorted square pyramidal environment, and two with one of the four copper atoms six-coordinated, being further linked to a triflate [Cu(2)] or a water molecule [Cu(11)] (Figs. S2-S47). In both compounds each copper atom is coordinated by a bpy ligand and linked by two μ_4-phosphate groups. A hydroxide group furnishes further bridging between Cu(1) and Cu(2) in $\mathbf{1^P}$ and Cu(1) and Cu(2), Cu(6) and Cu(7) and Cu(11) and Cu(12) in $\mathbf{2^M}$. N_2O_4 coordination chromophore about Cu(1)/$\mathbf{1^P}$ [or Cu(2) and Cu(11) in $\mathbf{2^M}$] and N_2O_3 of Cu(2) /$\mathbf{1^P}$ [or Cu(1), Cu(6), Cu(7) and Cu(12) in $\mathbf{2^M}$] are comprised of two bpy nitrogen donors, a phosphate oxygen atom, with corner shared μ_2-OH_{oxygen} atom completing the basal plane. Apical coordination is provided by the two bridging phosphate oxygen atoms, respectively. For Cu(1) in $\mathbf{1^P}$ a further coordination of a perchlorate anion in a monodentate fashion complete the coordination sphere. In $\mathbf{2^M}$ the coordination sphere around Cu(2) and Cu(11) is completed by a a further coordination of a triflate and a water molecule, respectively (see experimental structural section for details). The N_2O_3 coordination chromophore about Cu(3) and Cu(4) $\mathbf{1^P}$ [or Cu(3) and Cu(4), Cu(8) and Cu(9), Cu(13) and Cu(14) in $\mathbf{2^M}$] is provided by two bpy nitrogen donors and two phosphate oxygen atoms, which form the basal plane of the square-pyramid, with a coordinated water molecule occupying the apex of the pyramid.

Bonding about each copper centre is as expected for square pyramidal geometry [or octahedral for Cu(1) ($\mathbf{1^P}$) and Cu(2) and Cu(11) ($\mathbf{2^M}$)] and it varies with donor type and position with apical bond lengths significantly longer than the basal-plane/equatorial ones, as might be anticipated by the Jahn‑Teller theorem (Table S2-S5). Bonding about P atoms are consistent with the single and double bond nature of the coordinated and non-coordinated oxygen atoms, and the bridging mode disposes the four copper atoms in a butterfly arrangement with a $[Cu_4(\mu_4\text{-}PO_4)_2(\mu_2\text{-}OH)]$ core. Bridging between copper atoms through the hydroxide oxygen atom subtend angles of 114.0(4) in $\mathbf{1^P}$ and 112.0(4), 112.8(4) and 115.7(4)

2^{M} [for Cu(1)···Cu(2), Cu(6)···Cu(7) and Cu(11)···Cu(12). These bridging interactions dispose the four copper atoms of each core in an irregular trapezoidal arrangement. The bpy ligands of the cores participate in intramolecular offset π-π interactions and a separation at closest contact of 3.30 and 3.45 Å.

In the crystal lattice of **1^{P}** and **2^{M}**, there is a columnar staggered alignment of the cationic chains developing along the crystallographic *a* (**1^{P}**) and *b* axis (**2^{M}**) (Figure S5). The cohesion of adjacent chains is ensured by an extended network of H-bonds involving coordinated and non-coordinated anions and water molecules (see Table S2H for **1^{P}**) together with intermolecular π– π interactions that occur among the bpy ligands from the *connectors* and their related neighbors of the tetranuclear cores, giving closest contact separations varying in the ranges of 3.25–3.36 (**1^{P}**) and 3.36–3.48 Å (**2^{M}**).

Structural details for the tetrameric cores in compounds

$[Cu_5(bpy)_5(OH)(H_2O)_2(CMP)_2(ClO_4)](ClO_4)_4 \cdot 9H_2O$ (**1^P**) and

$\{[Cu_{15}(bpy)_{15}(OH)_3(H_2O)_{6.7}(CMP)_6(CF_3SO_3)_{1.3}](CF_3SO_3)_{13.7}\cdot 15H_2O\}_n$ (**2^M**)

(**1^P**)

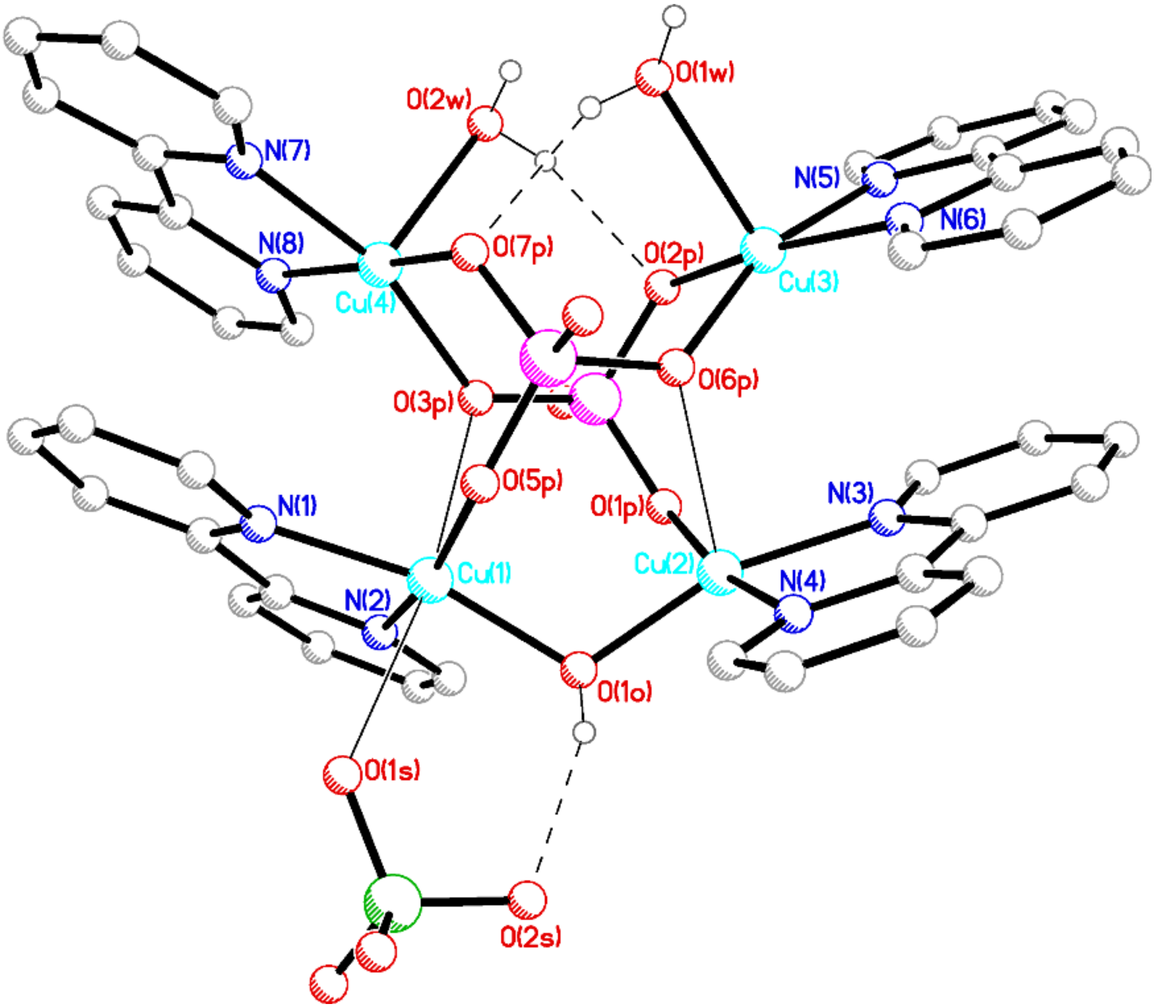

Fig. S1 Perspective view of $[Cu_4(bipy)_4(H_2O)_2(OH)(CMP)_2(ClO_4)]^{2+}$ *tetranuclear core* in **1^P**. (Cytosine nucleobase and sugar moiety have been omitted for clarity)

Table S2.

Selected bond distances [Å] for compound 1^{P}.

Cu(1)-N(1)	1.985(4)	Cu(2)-O(1O)	1.897(3)
Cu(1)-N(2)	2.004(4)	Cu(2)-N(3)	1.984(4)
Cu(1)-O(1O)	1.920(3)	Cu(2)-N(4)	1.990(3)
Cu(1)-O(3P)	2.515(3)	Cu(2)-O(1P)	1.931(3)
Cu(1)-O(5P)	1.958(3)	Cu(2)-O(6P)	2.488(3)
Cu(1)-O(1S)	2.778(4)		
Cu(3)-O(1w)	2.307(3)	Cu(4)-O(2w)	2.326(3)
Cu(3)-N(5)	1.992(3)	Cu(4)-N(7)	1.997(4)
Cu(3)-N(6)	2.003(3)	Cu(4)-N(8)	2.000(3)
Cu(3)-O(2P)	1.947(3)	Cu(4)-O(3P)	1.964(3)
Cu(3)-O(6P)	1.964(3)	Cu(4)-O(7P)	1.939(3)

Selected bond angles [^{o}] for compound 1^{P}.

O(1O)-Cu(1)-N(1)	166.94(13)	O(1O)-Cu(2)-N(3)	162.04(14)
O(1O)-Cu(1)-N(2)	94.12(14)	O(1O)-Cu(2)-N(4)	99.26(14)
O(1O)-Cu(1)-O(3P)	103.91(11)	O(1O)-Cu(2)-O(1P)	88.87(12)
O(1O)-Cu(1)-O(5P)	93.34(12)	O(1O)-Cu(2)-O(6P)	109.51(12)
O(1O)-Cu(1)-O(1S)	84.96(12)	N(3)-Cu(2)-N(4)	81.53(15)
N(1)-Cu(1)-N(2)	80.76(15)	N(3)-Cu(2)-O(1P)	92.97(13)
N(1)-Cu(1)-O(3P)	87.22(12)	N(3)-Cu(2)-O(6P)	88.44(12)
N(1)-Cu(1)-O(5P)	94.23(14)	N(4)-Cu(2)-O(1P)	169.12(13)
N(1)-Cu(1)-O(1S)	84.42(13)	N(4)-Cu(2)-O(6P)	83.82(12)
N(2)-Cu(1)-O(3P)	80.72(12)	O(1P)-Cu(2)-O(6P)	86.67(10)
N(2)-Cu(1)-O(5P)	166.16(13)		
N(2)-Cu(1)-O(1S)	102.36(15)		
O(3P)-Cu(1)-O(1S)	170.48(11)		
O(3P)-Cu(1)-O(5P)	86.18(10)		
O(5P)-Cu(1)-O(1S)	89.89(13)		
O(1w)-Cu(3)-N(5)	100.81(13)	O(2w)-Cu(4)-N(7)	99.22(13)
O(1w)-Cu(3)-N(6)	94.92(13)	O(2w)-Cu(4)-N(8)	89.25(13)
O(1w)-Cu(3)-O(2P)	94.85(12)	O(2w)-Cu(4)-O(3P)	96.14(12)
O(1w)-Cu(3)-O(6P)	96.46(12)	O(2w)-Cu(4)-O(7P)	96.58(12)
N(5)-Cu(3)-N(6)	81.05(15)	O(3P)-Cu(4)-O(7P)	92.05(12)
N(5)-Cu(3)-O(2P)	90.11(14)	N(7)-Cu(4)-N(8)	81.32(14)
N(5)-Cu(3)-O(6P)	162.29(13)	N(7)-Cu(4)-O(3P)	163.72(13)
N(6)-Cu(3)-O(2P)	167.90(14)	N(7)-Cu(4)-O(7P)	91.54(13)
N(6)-Cu(3)-O(6P)	93.64(13)	N(8)-Cu(4)-O(3P)	93.55(13)
O(2P)-Cu(3)-O(6P)	92.34(11)	N(8)-Cu(4)-O(7P)	171.45(13)

Table S2H. Hydrogen bonds [Å and °] for compound 1^{P}. [*a*]

D-H···A	d(D-H)	d(H···A)	d(D···A)	<(DHA)
O(1w)-H(1wA)···O(7w)	0.832(14)	1.998(16)	2.828(5)	176(5)
O(1w)-H(1wB)···O(7P)	0.838(14)	2.04(2)	2.815(4)	153(5)
O(2w)-H(2wB)···O(2P)	0.845(14)	2.02(3)	2.784(4)	150(5)
O(1O)-H(1O)···O(2S)	0.796(14)	2.15(2)	2.907(5)	159(5)
N(4A)-H(4NA)···O(2B)#1	0.78(2)	2.37(4)	3.055(5)	147(5)
N(4A)-H(4NB)···O(8w)	0.80(2)	2.18(4)	2.886(6)	147(4)
O(2'A)-H(2OA)···O(10w)	0.84	2.39	3.130(7)	147.4
O(3'A)-H(3OA)···O(7S)	0.84	2.06	2.867(5)	160.6
N(4B)-H(4NC)···O(2A)#2	0.79(2)	2.14(3)	2.883(5)	155(5)
N(4B)-H(4ND)···O(10w)#2	0.81(2)	1.96(3)	2.759(6)	166(6)
O(2'B)-H(2OB)···O(19S)#2	0.84	2.19	2.944(4)	149.2
O(3'B)-H(3OB)···O(4w)#2	0.84	1.90	2.724(5)	166.4
O(3w)-H(3wA)···O(8S)	0.836(14)	2.17(2)	2.973(6)	162(6)
O(3w)-H(3wB)···O(11w)	0.836(14)	1.85(2)	2.668(6)	166(7)
O(4w)-H(4wA)···O(6w)	0.839(14)	1.962(17)	2.793(6)	171(6)
O(4w)-H(4wB)···O(1S)#1	0.839(14)	2.27(3)	3.075(6)	161(7)
O(5w)-H(5wA)···O(3'A)#3	0.839(14)	1.96(2)	2.788(5)	167(6)
O(5w)-H(5wB)···O(17S)	0.838(14)	1.98(2)	2.779(6)	159(6)
O(6w)-H(6wA)···O(2S)#4	0.840(14)	2.30(4)	3.079(6)	153(7)
O(6w)-H(6wB)···O(12S)	0.843(14)	2.031(16)	2.870(6)	174(6)
O(7w)-H(7wA)···O(13S)#2	0.846(14)	2.12(3)	2.898(5)	152(6)
O(7w)-H(7wB)···O(10w)#2	0.847(14)	1.99(3)	2.807(6)	160(7)
O(8w)-H(8wA)···O(14S)	0.830(14)	2.16(3)	2.957(6)	160(8)
O(8w)-H(8wB)···O(11S)	0.835(14)	2.12(2)	2.941(7)	169(8)
O(9w)-H(9wA)···O(12S)	0.852(14)	2.030(18)	2.871(7)	169(6)
O(9w)-H(9wB)···O(7w)	0.847(14)	1.88(2)	2.712(7)	167(7)
O(10w)-H(10A)···O(3w)	0.846(15)	1.98(4)	2.740(6)	150(7)
O(11w)-H(11A)···O(5w)#5	0.850(14)	1.92(4)	2.728(5)	160(9)
O(11w)-H(11B)···O(3'B)#6	0.849(14)	2.01(3)	2.819(5)	160(9)
O(4w)-H(4wB)···Cl(1)#1	0.839(14)	2.964(19)	3.788(4)	168(6)
O(4w)-H(4wB)···O(3S)#1	0.839(14)	2.59(5)	3.282(7)	141(6)

[a] Symmetry transformations used to generate equivalent atoms: #1 *x*+1/2,-*y*+3/2,-*z*+2
#2 *x*-1/2,-*y*+3/2,-*z*+2 #3 -*x*+2,*y*-1/2,-*z*+5/2 #4 *x*,*y*-1,*z* #5 *x*+1,*y*,*z* #6 -*x*+5/2,-*y*+2,*z*+1/2

2. {[$Cu_{15}(bpy)_{15}(OH)_3(H_2O)_{6.7}(CMP)_6(CF_3SO_3)_{1.3}](CF_3SO_3)_{13.7}\cdot 15H_2O$}$_n$ (**2^M^**)

There are 3 tetrameric clusters in this compound:

CLUSTER I

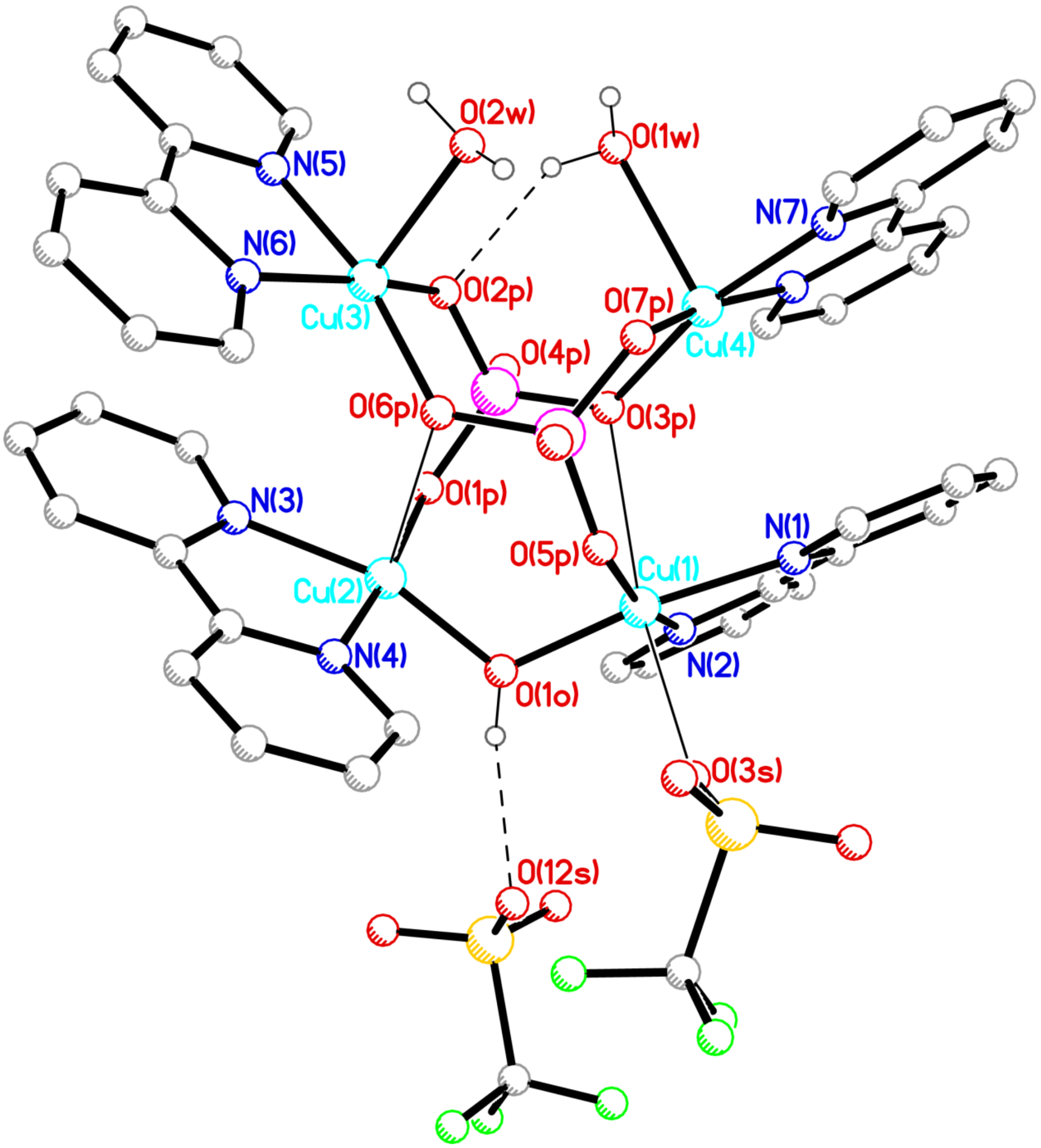

Fig. S2. Perspective view of $[Cu_4(bpy)_4(OH)(H_2O)_2(CMP)_2(CF_3SO_3)]^{2+}$ *tetranuclear core* and non-coordinated $CF_3SO_3^-$ anion H-bonded to hydroxo group in **2^M^**. (Cytosine nucleobase and sugar moiety have been omitted for clarity).

Table S3.

Selected bond distances [Å] in cluster I for compound 2[M].

Cu(1)-O(1O)	1.930(4)	Cu(2)-O(1O)	1.913(4)
Cu(1)-O(5P)	1.940(3)	Cu(2)-O(1P)	1.948(3)
Cu(1)-N(1)	2.005(2)	Cu(2)-N(4)	1.976(2)
Cu(1)-N(2)	2.007(2)	Cu(2)-N(3)	2.016(2)
Cu(1)-O(3S)	2.633(2)	Cu(2)-O(9S)	2.628(2)
Cu(1)-O(3P)	2.688(2)	Cu(2)-O(6P)	2.596(2)
Cu(3)-O(2P)	1.946(3)	Cu(4)-O(7P)	1.939(3)
Cu(3)-O(6P)	1.964(3)	Cu(4)-O(3P)	1.997(3)
Cu(3)-N(6)	1.978(3)	Cu(4)-N(8)	1.999(2)
Cu(3)-N(5)	1.999(3)	Cu(4)-N(7)	2.001(2)
Cu(3)-O(2W)	2.284(4)	Cu(4)-O(1W)	2.347(4)

Selected bond angles [^{o}] in cluster I for compound 2[M].

O(1O)-Cu(1)-O(5P)	91.41(16)	O(2P)-Cu(3)-O(6P)	95.34(15)
O(1O)-Cu(1)-N(1)	171.84(14)	O(2P)-Cu(3)-N(6)	161.84(15)
O(5P)-Cu(1)-N(1)	95.51(14)	O(6P)-Cu(3)-N(6)	92.87(15)
O(1O)-Cu(1)-N(2)	93.91(15)	O(2P)-Cu(3)-N(5)	88.28(15)
O(5P)-Cu(1)-N(2)	166.70(15)	O(6P)-Cu(3)-N(5)	168.58(15)
N(1)-Cu(1)-N(2)	80.27(13)	N(6)-Cu(3)-N(5)	80.76(15)
O(1O)-Cu(2)-O(1P)	87.84(16)	O(2P)-Cu(3)-O(2W)	103.35(16)
O(1O)-Cu(2)-N(4)	98.97(14)	O(6P)-Cu(3)-O(2W)	97.46(13)
O(1P)-Cu(2)-N(4)	171.73(15)	N(6)-Cu(3)-O(2W)	91.58(16)
O(1O)-Cu(2)-N(3)	159.77(15)	N(5)-Cu(3)-O(2W)	92.21(15)
O(1P)-Cu(2)-N(3)	94.35(14)	O(7P)-Cu(4)-O(3P)	94.10(13)
N(4)-Cu(2)-N(3)	80.90(12)	O(7P)-Cu(4)-N(8)	168.33(13)
		O(3P)-Cu(4)-N(8)	91.85(12)
		O(7P)-Cu(4)-N(7)	91.28(12)
		O(3P)-Cu(4)-N(7)	167.33(13)
		N(8)-Cu(4)-N(7)	80.97(11)
		O(7P)-Cu(4)-O(1W)	102.42(14)
		O(3P)-Cu(4)-O(1W)	97.65(12)
		N(8)-Cu(4)-O(1W)	86.70(13)
		N(7)-Cu(4)-O(1W)	92.37(13)
Cu(1)-O(1O)-Cu(2)	112.4(2)		

CLUSTER II

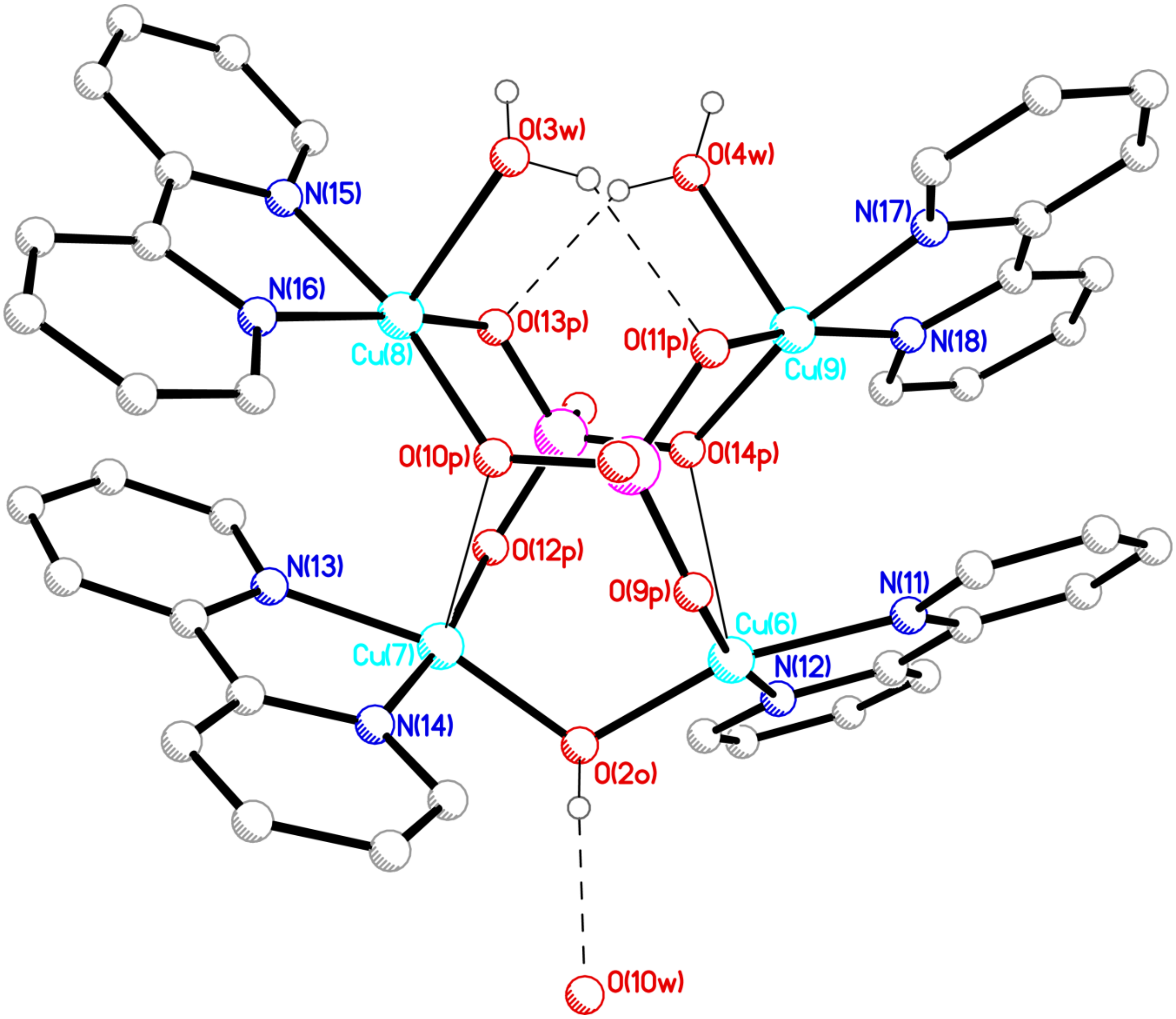

Fig. S3. Perspective view of $[Cu_4(bpy)_4(OH)(H_2O)_2(CMP)_2]^{3+}$ *tetranuclear core* containing only pentacoordinated copper ions in **2^M**. (Cytosine nucleobase and sugar moiety have been omitted for clarity).

Table S4.

Selected bond distances [Å] in cluster II for compound 2^M.

Cu(6)-O(2O)	1.921(4)	Cu(8)-O(14P)	1.962(3)
Cu(6)-O(9P)	1.937(3)	Cu(8)-O(10P)	1.978(3)
Cu(6)-N(12)	1.990(3)	Cu(8)-N(15)	2.002(3)
Cu(6)-N(11)	1.998(2)	Cu(8)-N(16)	2.005(2)
Cu(7)-O(2O)	1.900(4)	Cu(8)-O(3W)	2.282(4)
Cu(7)-O(13P)	1.955(3)	Cu(9)-O(11P)	1.968(3)
Cu(7)-N(13)	1.982(2)	Cu(9)-O(15P)	1.975(3)
Cu(7)-N(14)	1.987(2)	Cu(9)-N(17)	1.983(2)
Cu(6)-O(14P)	2.561(2)	Cu(9)-N(18)	2.011(2)
Cu(7)-O(10P)	2.558(2)	Cu(9)-O(4W)	2.275(4)

Selected bond angles [^{o}] in cluster II for compound 2^M.

O(2O)-Cu(6)-O(9P)	93.69(16)	O(14P)-Cu(8)-O(10P)	93.19(14)
O(2O)-Cu(6)-N(12)	93.52(15)	O(14P)-Cu(8)-N(15)	91.33(14)
O(9P)-Cu(6)-N(12)	170.87(15)	O(10P)-Cu(8)-N(15)	169.40(14)
O(2O)-Cu(6)-N(11)	164.64(15)	O(14P)-Cu(8)-N(16)	164.93(14)
O(9P)-Cu(6)-N(11)	93.63(14)	O(10P)-Cu(8)-N(16)	93.11(12)
N(12)-Cu(6)-N(11)	80.73(13)	N(15)-Cu(8)-N(16)	80.31(13)
O(2O)-Cu(7)-O(13P)	89.24(16)	O(14P)-Cu(8)-O(3W)	100.59(14)
O(2O)-Cu(7)-N(13)	160.62(16)	O(10P)-Cu(8)-O(3W)	95.05(12)
O(13P)-Cu(7)-N(13)	93.05(14)	N(15)-Cu(8)-O(3W)	93.53(15)
O(2O)-Cu(7)-N(14)	99.32(15)	N(16)-Cu(8)-O(3W)	92.47(14)
O(13P)-Cu(7)-N(14)	169.10(15)	O(11P)-Cu(9)-O(15P)	92.55(13)
N(13)-Cu(7)-N(14)	81.13(13)	O(11P)-Cu(9)-N(17)	91.44(13)
		O(15P)-Cu(9)-N(17)	169.30(15)
		O(11P)-Cu(9)-N(18)	163.95(13)
		O(15P)-Cu(9)-N(18)	93.06(12)
		N(17)-Cu(9)-N(18)	80.58(12)
		O(11P)-Cu(9)-O(4W)	101.68(14)
		O(15P)-Cu(9)-O(4W)	93.64(13)
		N(17)-Cu(9)-O(4W)	95.27(14)
		N(18)-Cu(9)-O(4W)	92.96(13)
Cu(7)-O(2O)-Cu(6)	113.06(19)		

CLUSTER III

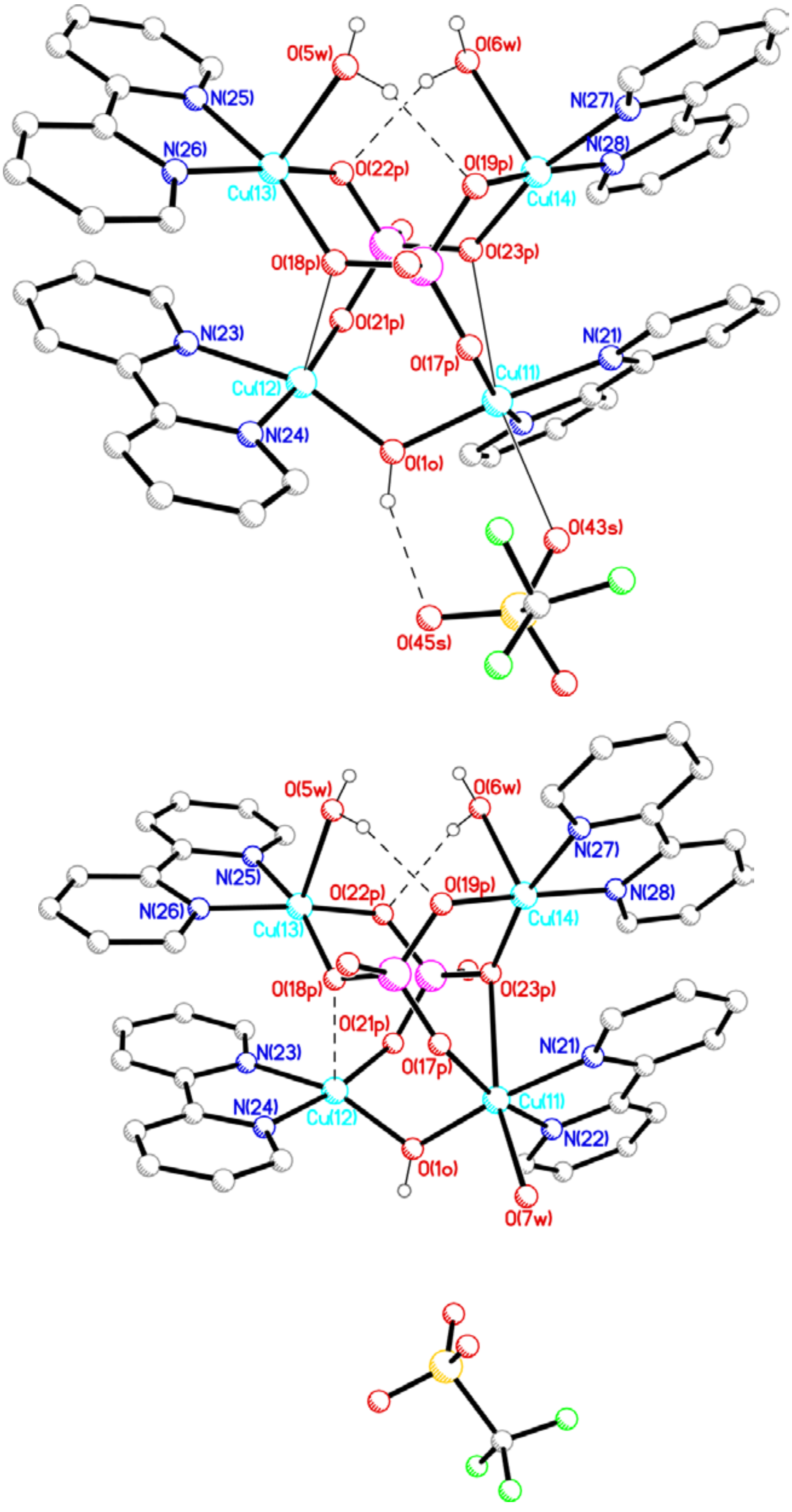

Fig. S4. Perspective view of $[Cu_4(bpy)_4(OH)(H_2O)_2(CMP)_2(CF_3SO_3)]^{2+}$ (top) or $[Cu_4(bpy)_4(OH)(H_2O)_3(CMP)_2]^{3+}$ *tetranuclear cores* (bottom) in **2^{M}** where the cytosine nucleobase and sugar moiety have been omitted for clarity. (The best structural model provided atom Cu(11) in cluster III being coordinated either to a water molecule or a triflate ion with a statistics of about 70% vs. 30%, respectively. See crystal structure experimental section, refinement details for **2^{M}**).

Table S5.

Selected bond distances [Å] in cluster III for compound 2^M.

Cu(11)-O(3O)	1.931(4)	Cu(13)-O(22P)	1.956(3)
Cu(11)-O(17P)	1.966(3)	Cu(13)-N(25)	1.987(2)
Cu(11)-N(22)	1.995(3)	Cu(13)-O(18P)	1.996(3)
Cu(11)-N(21)	2.005(2)	Cu(13)-N(26)	2.005(2)
Cu(11)-O(7W)	2.375(10)	Cu(13)-O(5W)	2.268(4)
Cu(12)-O(3O)	1.897(4)	Cu(14)-O(19P)	1.952(3)
Cu(12)-O(21P)	1.946(3)	Cu(14)-O(23P)	1.976(3)
Cu(12)-N(24)	1.984(2)	Cu(14)-N(28)	1.992(2)
Cu(12)-N(23)	2.019(2)	Cu(14)-N(27)	1.999(2)
Cu(11)-O(23P)	2.759(2)	Cu(14)-O(6W)	2.356(4)
Cu(12)-O(18P)	2.451(2)		

Selected bond angles [°] in cluster III for compound 2^M.

O(3O)-Cu(11)-O(17P)	92.07(15)	O(22P)-Cu(13)-N(25)	90.50(14)
O(3O)-Cu(11)-N(22)	92.70(15)	O(22P)-Cu(13)-O(18P)	93.12(13)
O(17P)-Cu(11)-N(22)	165.48(16)	N(25)-Cu(13)-O(18P)	166.24(14)
O(3O)-Cu(11)-N(21)	173.03(14)	O(22P)-Cu(13)-N(26)	165.44(14)
O(17P)-Cu(11)-N(21)	94.76(13)	N(25)-Cu(13)-N(26)	80.58(13)
N(22)-Cu(11)-N(21)	81.00(13)	O(18P)-Cu(13)-N(26)	93.04(13)
O(3O)-Cu(11)-O(7W)	92.7(3)	O(22P)-Cu(13)-O(5W)	99.40(14)
O(17P)-Cu(11)-O(7W)	92.6(3)	N(25)-Cu(13)-O(5W)	99.79(14)
N(22)-Cu(11)-O(7W)	100.8(3)	O(18P)-Cu(13)-O(5W)	92.72(12)
N(21)-Cu(11)-O(7W)	85.6(3)	N(26)-Cu(13)-O(5W)	93.48(13)
O(3O)-Cu(12)-O(21P)	89.34(16)	O(19P)-Cu(14)-O(23P)	94.20(13)
O(3O)-Cu(12)-N(24)	97.86(15)	O(19P)-Cu(14)-N(28)	169.17(13)
O(21P)-Cu(12)-N(24)	171.08(14)	O(23P)-Cu(14)-N(28)	92.20(12)
O(3O)-Cu(12)-N(23)	158.85(15)	O(19P)-Cu(14)-N(27)	91.05(13)
O(21P)-Cu(12)-N(23)	94.55(13)	O(23P)-Cu(14)-N(27)	166.83(14)
N(24)-Cu(12)-N(23)	80.63(12)	N(28)-Cu(14)-N(27)	80.91(12)
		O(19P)-Cu(14)-O(6W)	100.02(15)
		O(23P)-Cu(14)-O(6W)	93.59(13)
		N(28)-Cu(14)-O(6W)	88.27(14)
Cu(12)-O(3O)-Cu(11)	116.13(18)	N(27)-Cu(14)-O(6W)	97.38(14)

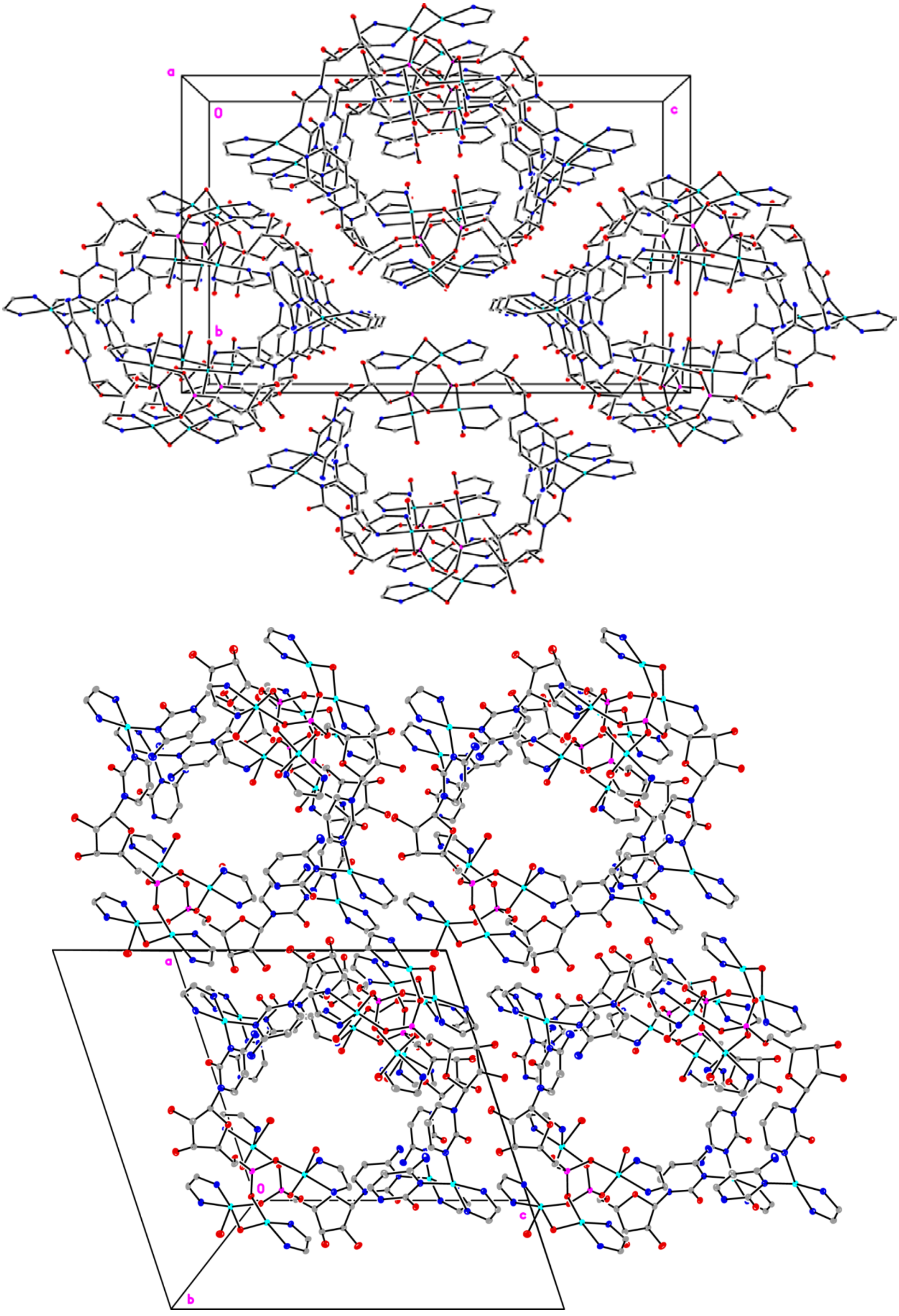

Fig. S5. View normal to (100) for $\mathbf{1^{P}}$ (top) and to (010) for $\mathbf{2^{M}}$ (bottom) helices.

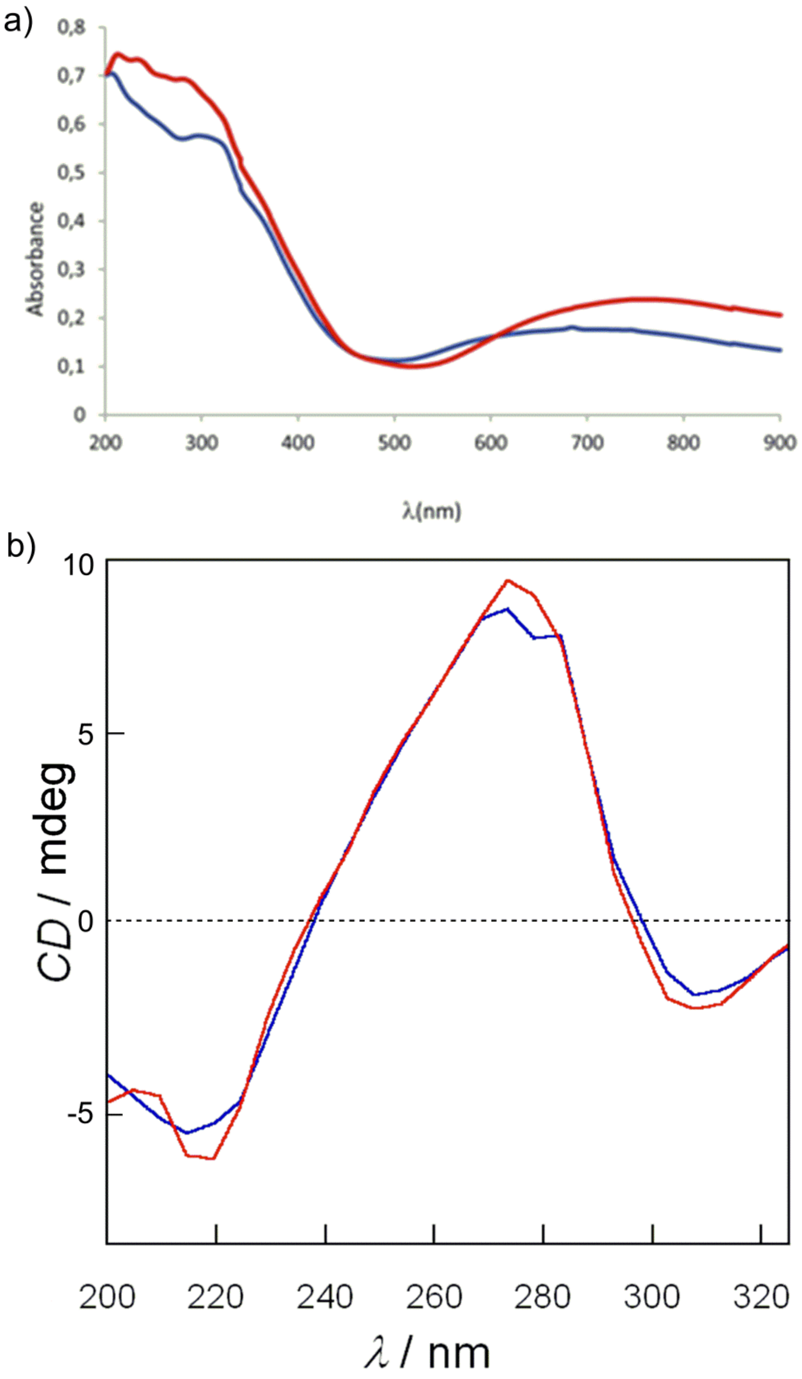

Fig. S6 (a) UV-Vis absorption spectra of solid samples $\mathbf{1}^{\mathbf{P}}$ (blue) and $\mathbf{2}^{\mathbf{M}}$ (red). (b) CD spectra showing identical Cotton effects for both $\mathbf{1}^{\mathbf{P}}$ and $\mathbf{2}^{\mathbf{M}}$ in the UV region, that is, negative and positive Cotton effects at *ca*. 220 and 280 nm, respectively.

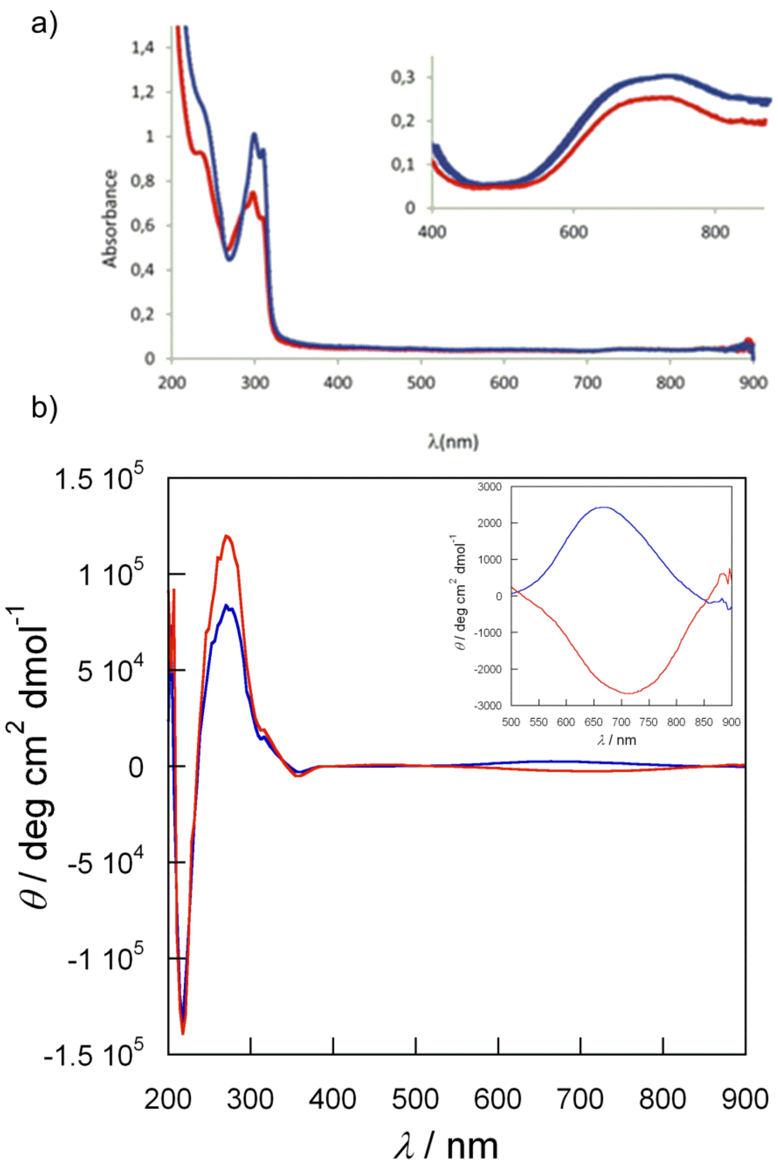

Fig. S7 . (a) UV-Vis spectra of $\mathbf{1^P}$ (blue) and $\mathbf{2^M}$ (red) in H_2O/CH_3CN (1:1 v:v) solution at 1×10^{-5} mol·L^{-1}. The inset shows the visible spectra of $\mathbf{1^P}$ (blue) and $\mathbf{2^M}$ (red) in H_2O/CH_3CN (1:1 v:v) solution at 1×10^{-3} mol·L^{-1}. (b) CD spectra of $\mathbf{1^P}$ (blue) and $\mathbf{2^M}$ (red) in H_2O/CH_3CN (1:1 v:v) solution. The inset shows in detail the Cotton effects corresponding to the copper(II) ions.

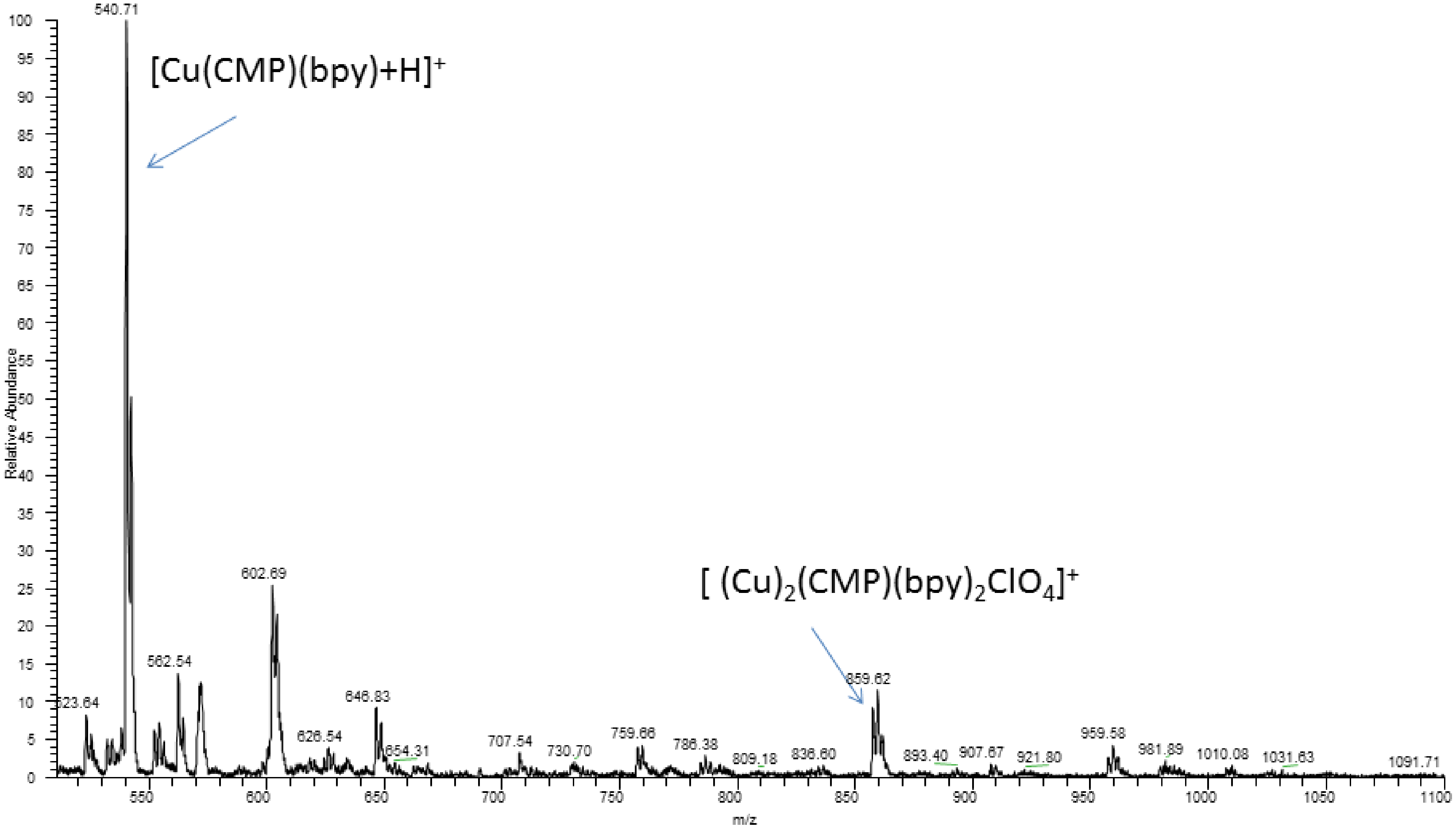

Fig. S8a. Experimental ESI(+)-MS spectrum of a **1P** H_2O/CH_3CN (1:1 v:v) solution.

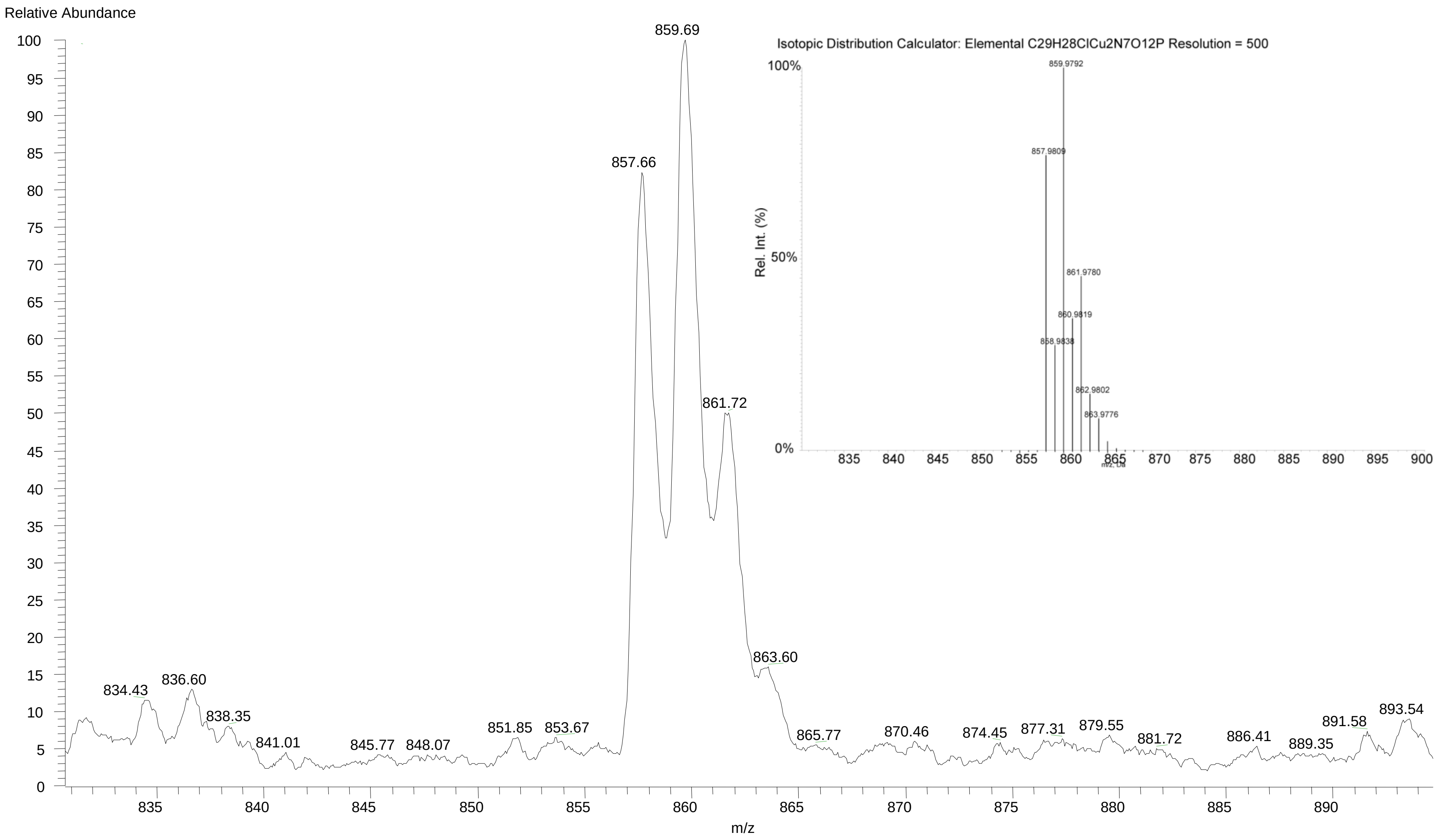

Fig. S8b. Experimental ESI(+)-MS spectrum of a **1^P^** H_2O/CH_3CN (1:1 v:v) solution with calculated isotopic pattern (upper right inset) for peak at *m/z* 859.69.

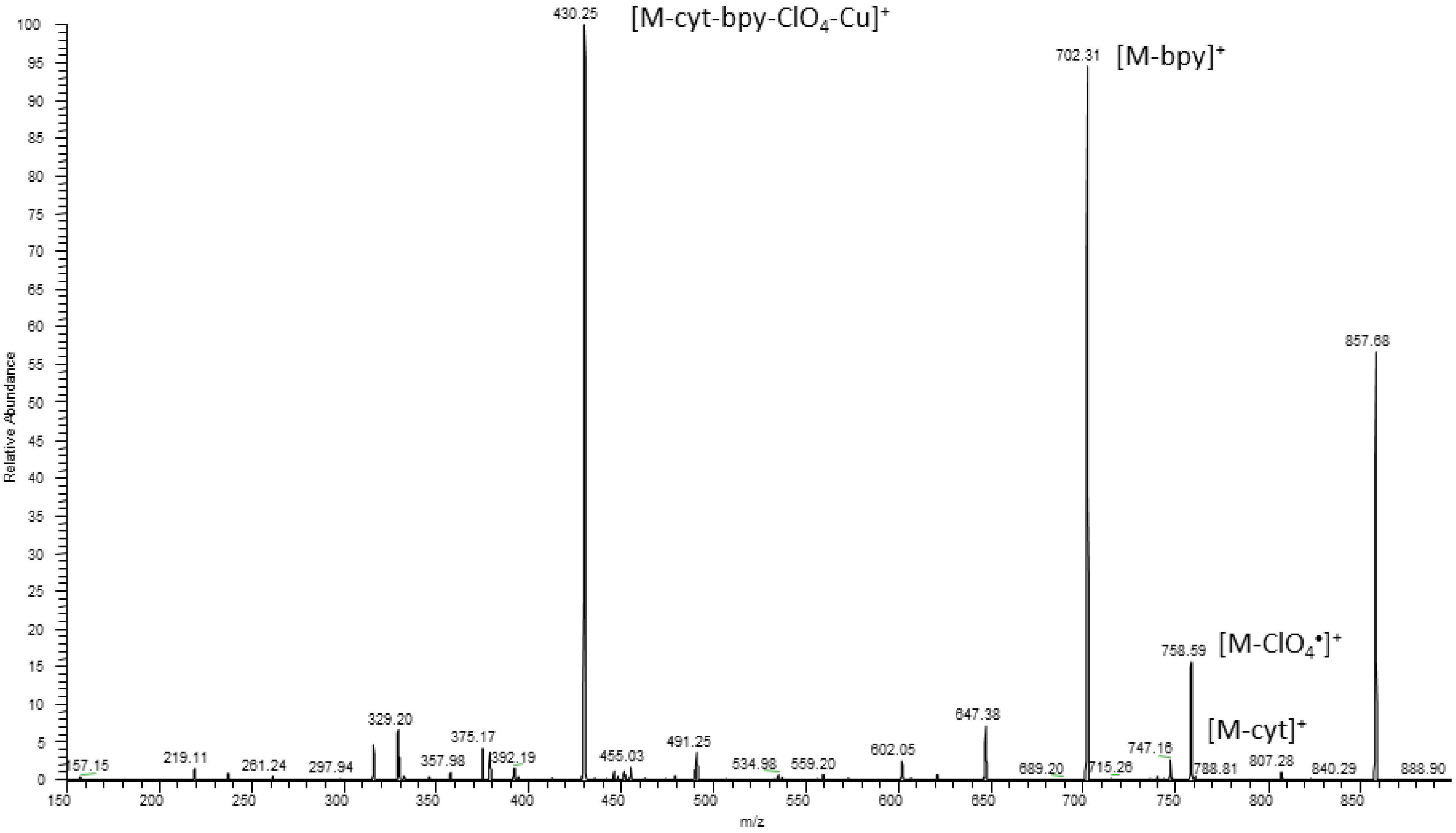

Fig. S9 ESI(+)-MS/MS spectrum performed on peak at *m/z* 857.7 (see Fig. S8a) revealing the fragmentation pattern of the $[Cu_2(CMP)(bpy)_2(ClO_4)]^+$ ion upon collision-induced dissociation. The most abundant fragment is the monomeric species $[Cu(CMP\text{-}cyt)(bpy)]^+$ (wherein the breaking of the N-glycosidic bond in CMP had occurred), while the second most abundant is the dimeric $[Cu_2(CMP)(bpy)(ClO_4)]^+$ ion.

.

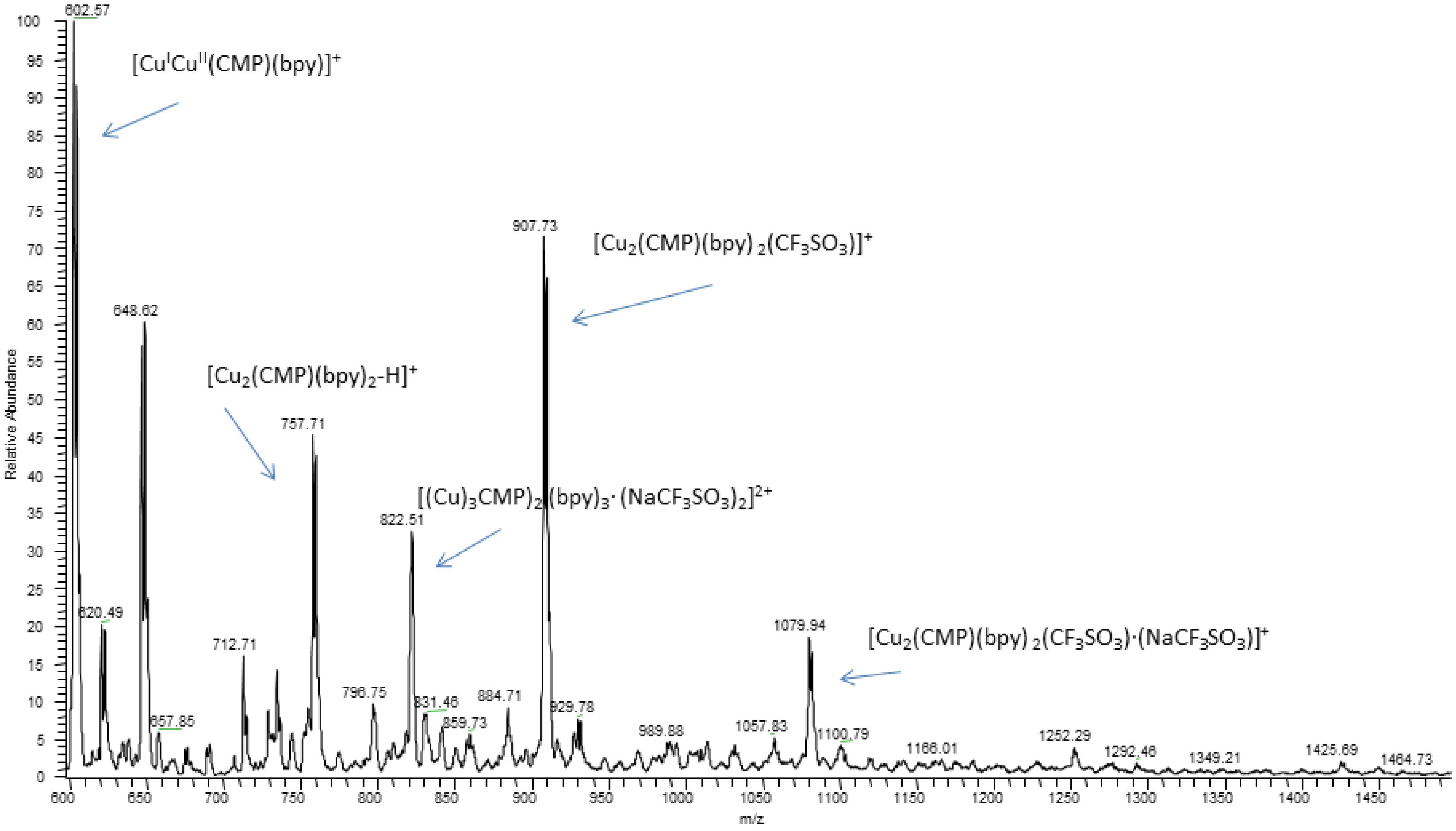

Fig. S10. Experimental ESI(+)-MS spectrum of a **2ᴹ** H_2O solution.

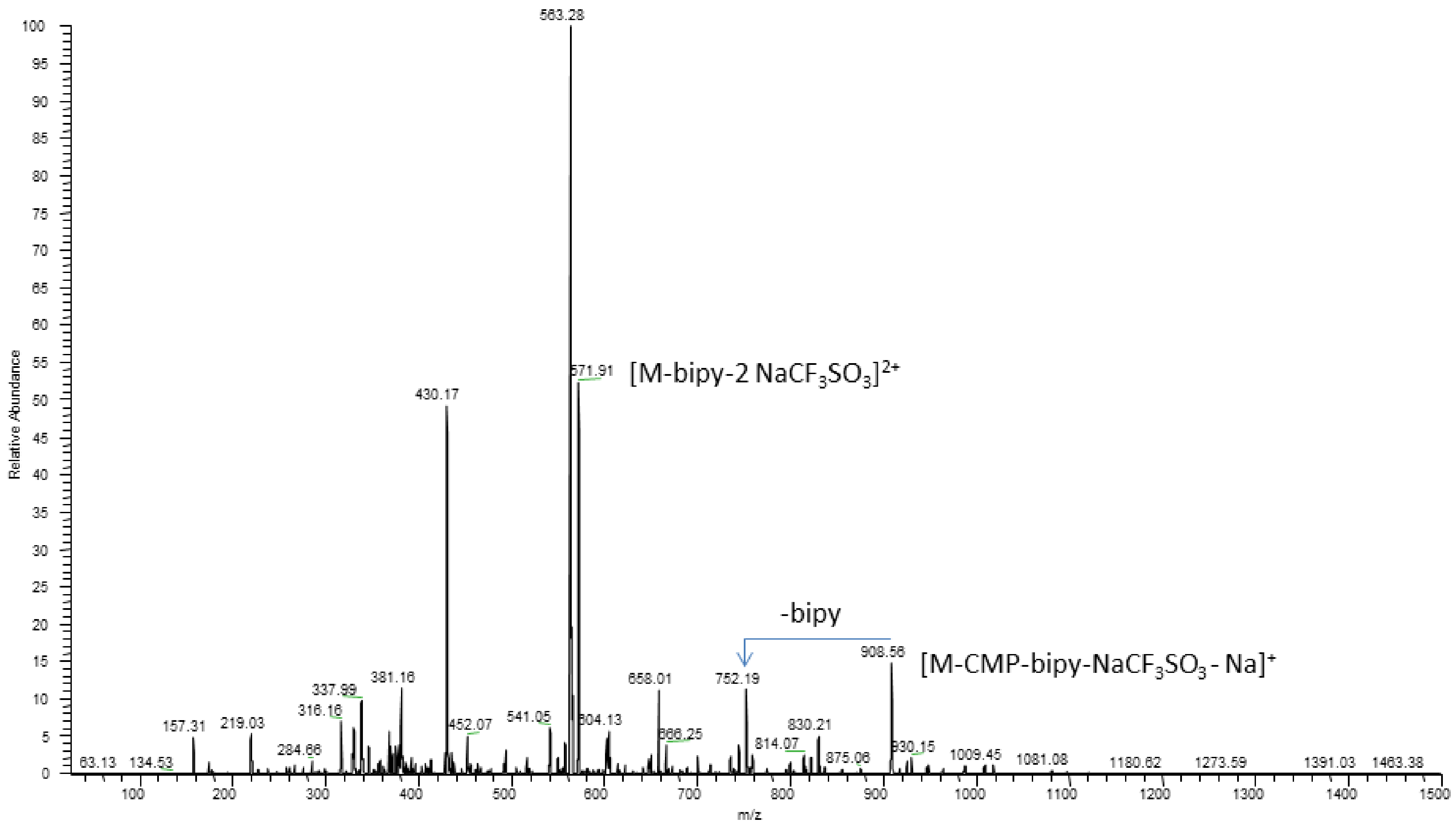

Figure S11. ESI(+)-MS/MS spectrum performed on double charged peak at *m/z* 822.5 (see Fig. S10) revealing the fragmentation pattern of the $[Cu_3(CMP)_2(bpy)_3{\cdot}(NaCF_3SO_3)_2]^{2+}$ ion upon collision-induced dissociation. Three trimeric species are revealed at m/z 908.5, 752.2, and 571.9.

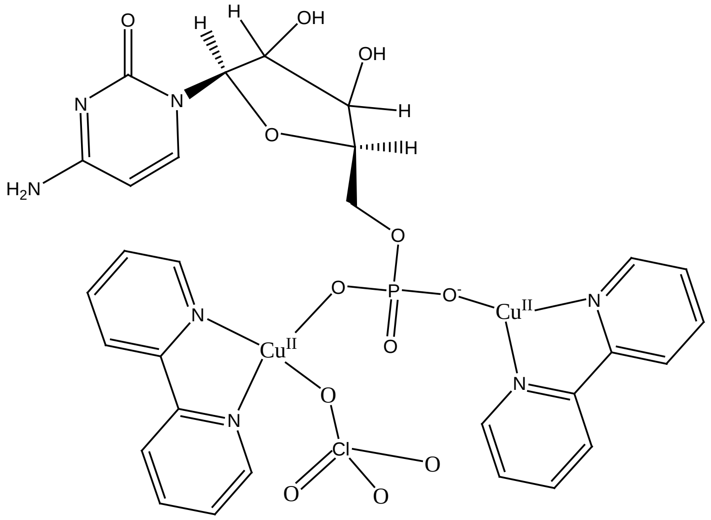

A half of the *tetranuclear core*

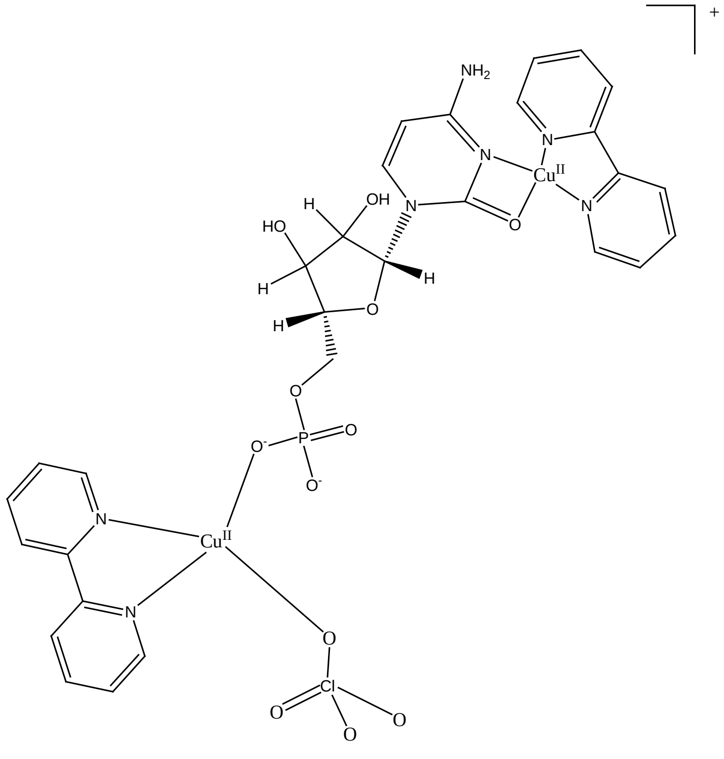

A fragment of *tetranuclear core* and *connector*.

Scheme S1. Alternative hypotheses for the ESI(+)-MS ion at *m/z* 857.7 in H_2O/CH_3CN (1:1 v:v) solution of **1**$^{\mathbf{P}}$.

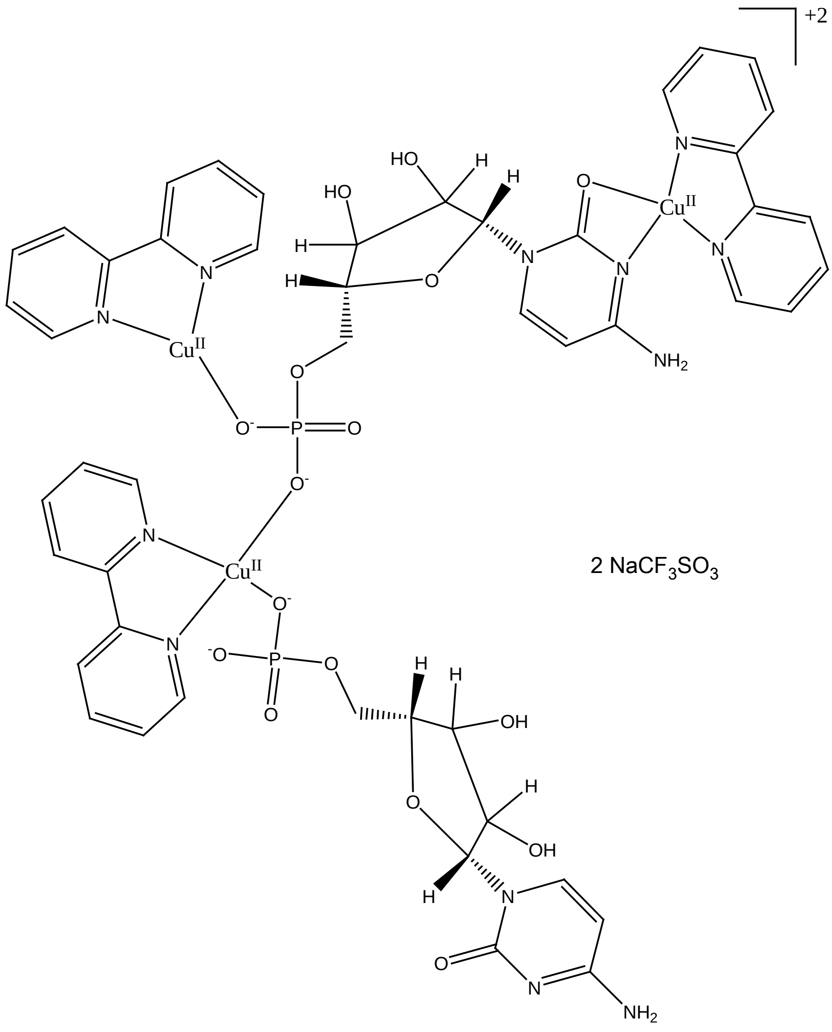

Scheme S2. Proposed structure for the *m*/*z* 822.5 ion in H_2O solution of **2^M^**. (The presence of $NaCF_3SO_3$ salt in the composition of the ESI(+)-MS peak is likely due to impurities of dissolved solid).

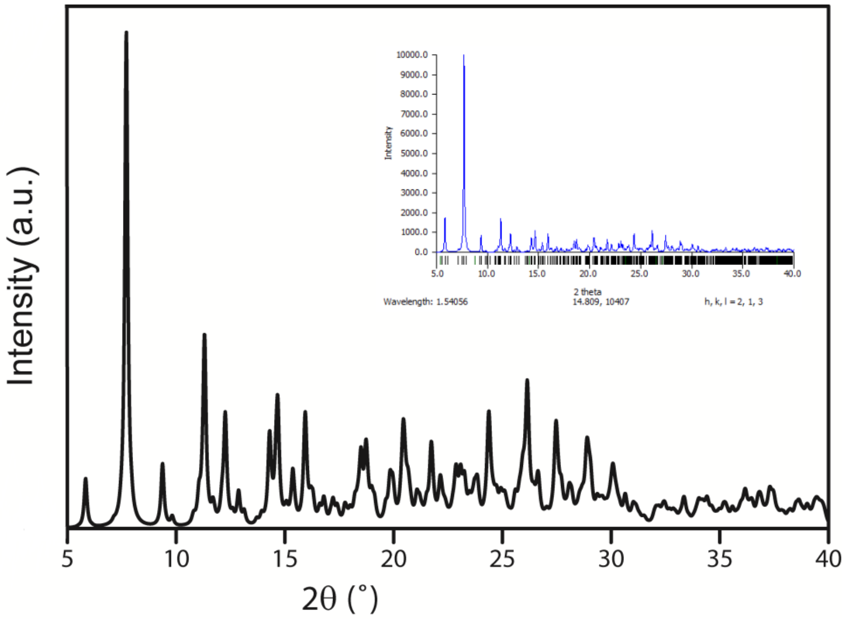

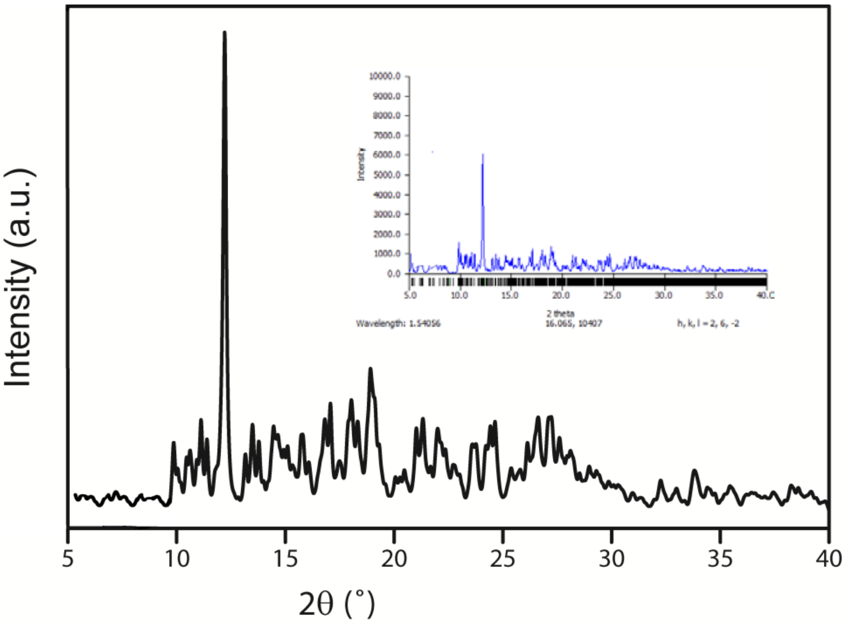

Fig. S12 Experimental XRPD pattern profiles of **1^P^** (up) and **2^M^** (bottom), measured in the 2θ range 5.0–40.0° at r.t. on powder samples obtained upon metathetical exchange in water of **2^M^** with $LiClO_4$ (up) and **1^P^** with $LiCF_3SO_3$ (bottom). The insets show the calculated XRPD pattern profiles of the crystalline phases of **1^P^** (up), and **2^M^** (bottom).
